# Supplementary material for: Alcohol and the risk of pneumonia: a systematic review and meta-analysis
Source: BMJ Open. 2018 Aug 22;8(8):e022344. doi: 10.1136/bmjopen-2018-022344 (PMC6112384; doi:10.1136/bmjopen-2018-022344)
Supplement: Supplementary file 1 [file bmjopen-2018-022344supp001.pdf]

Alcohol and the risk of pneumonia: A systematic review and meta-analysis

Evangelia Simou, John Britton, Jo Leonardi-Bee

Online Data Supplement

**Table E1: Medline (via Ovid) and EMBASE (via Ovid) search terms for primary studies**	
**Medline via Ovid search terms**	
1.	Epidemiologic studies/
2.	Exp case control studies/
3.	Exp cohort studies/
4.	Case control.tw.
5.	(cohort adj (study or studies)).tw.
6.	Cohort analy$.tw.
7.	(Follow up adj (study or studies)).tw.
8.	(observational adj (study or studies)).tw.
9.	Longitudinal.tw.
10.	Retrospective.tw.
11.	Cross sectional.tw.
12.	Cross-sectional studies/
13.	Or/1-12
14.	exp Alcohol-Related Disorders/
15.	Alcohol Drinking/
16.	(alcohol adj3 (drink$ or intoxicat$ or use$ or abus$ or misus$ or risk$ or consum$ or withdraw$ or detox$ or treat$ or therap$ or excess$ or reduc$ or cessation or intervention$)).tw.
17.	(drink$ adj3 (excess or heavy or heavily or harm or harmful or hazard$ or binge or problem$)).tw.
18.	alcoholic$.tw.
19.	14 or 15 or 16 or 17 or 18
20.	exp Respiratory Tract Infections/
21.	acute respiratory infection*.tw.
22.	lower respiratory infection*.tw.
23.	lower respiratory tract infection*.tw.
24.	exp Pneumonia/
25.	(pneumon* or bronchopneumon* or pleuropneumon*).tw.
26.	exp Bronchitis/
27.	(bronchit* or bronchiolit*).tw.
28.	20 or 21 or 22 or 23 or 24 or 25 or 26 or 27
29.	13 and 19 and 28
**Embase via Ovid search terms**	
1.	Clinical study/
2.	Case control study
3.	Family study/
4.	Longitudinal study/
5.	Retrospective study/
6.	Prospective study/
7.	Randomized controlled trials/
8.	6 not 7
9.	Cohort analysis/
10.	(Cohort adj (study or studies)).mp.
11.	(Case control adj (study or studies)).tw.
12.	(follow up adj (study or studies)).tw.
13.	(observational adj (study or studies)).tw.
14.	(epidemiologic$ adj (study or studies)).tw.
15.	(cross sectional adj (study or studies)).tw.
16.	Or/1-5,8-15
17.	substance-related disorders/
18.	((drug or substance) adj (Addict$ or abus$ or dependen$)).mp
19.	(intoxicat$ or abstinen$ or withdrawal$).mp.
20.	(excessive$ adj use$).mp.
21.	(use$ adj disorder$).mp.

22.	(drinking adj behavi$3).mp.
23.	drinking behavior.mp.
24.	alcohol$.mp.
25.	alcoholism/
26.	(alcohol adj abuse).mp
27.	17 or 18 or 19 or 20 or 21 or 22 or 23 or 24 or 25 or 26
28.	*PNEUMONIA/
29.	bacterial pneumonia/ or infectious pneumonia/
30.	Mycoplasma pneumonia/
31.	COMMUNITY ACQUIRED PNEUMONIA/
32.	mycoplasma pneumon*.tw.
33.	(community-acquired pneumon* or community acquired pneumon*).tw.
34.	28 or 29 or 30 or 31 or 32 or 33
35.	16 and 27 and 34
**Web of Science search terms**	
(alcohol* OR alcoholic beverage OR alcohol consumption OR alcohol drinking OR alcohol use OR alcohol intake OR alcoholism OR alcohol abuse OR ethanol* OR ethanol concentration) AND (Pneumonia OR pneumon* OR bronchopneumon* OR bronchitis) AND (longitudinal * OR case control* OR Cohort* OR case-control OR observational)	

Table E2: Data extraction form

Reviewer name: **Study Author and Year:**

DESCRIPTION OF STUDY

Study Design	Cohort ☐ Prospective ☐ Retrospective ☐ Nested Case control ☐
Name of Cohort<br><br>Data collection years	Start date (year): End date (year):<br><br>Years of follow-up data:
Definition of Alcohol<br><br>(Any record of the number of drinks per day or gr of ethanol per day, number of drinks consumed annually, record of drinking levels: light, moderate and heavy drinking age since started alcohol consumption, specific alcohol drinks, alcoholism)	
Definition of health condition	Method of diagnosis:<br>Exposure Outcome Both
Setting (e.g. developed/non-developed, public/private health care, urban/rural)	
Country- European?	
Selection of controls	

(for nested case control studies only)	

PARTICIPANTS

Inclusion criteria	
Exclusion criteria	
Number entering into study (may not be recorded)	
Final number of participants evaluated	

DEMOGRAPHICS OF STUDY POPULATION

Age (mean, SD, range)	
Gender (n, % male)	
Other demographics reported (e.g. ethnicity, place of residence, occupation, education, socioeconomic)	

RESULTS

Outcome	Adjusted measure of effect with 95% CI (in preference)<br>Crude measure of effect with 95% CI<br>Raw numbers<br>P value
1.first outcome	Exposure:<br>Comparator:<br>Result:
2. second outcome	Exposure:<br>Comparator:<br>Result:

SOURCE OF FUNDING

(e.g. Government (NHS), voluntary/charity, pharmaceutical company)

LIMITATIONS

Identified by author	
Identified by review team	
Evidence gaps and/or recommendations for future research	

Table E3: Exploration of heterogeneity for alcohol consumption and CAP risk

Factor	Number of studies	Pooled RR (95% CI)	I^2	P value for subgroup differences
**Overall result**	14	1.83 [1.30, 2.57]	91%	-
**Study design**				0.07
Case control	9	2.16 [1.64, 2.85]	71%	
Cohort	4	1.56 [0.84, 2.91]	92%	
Cross sectional	1	1.20 [0.77, 1.85]	-	
**Methodological quality**				0.09
High quality (>6)	8	2.20 [1.40, 3.47]	93%	
Low quality (<6)	6	1.36 [0.99, 1.87]	57%	
**Alcohol consumption**				0.39
Alcohol vs no alcohol	6	1.61 [1.25, 2.08]	25%	
Alcohol vs lowest category of exposure	6	2.07 [1.24, 3.44]	95%	
**CAP ascertainment**				0.002
Clinical diagnosis	11	1.81[1.25, 2.61]	81%	
Death records	1	3.33 [2·92, 3.79]	0%	
**Geographic location**				0.0003
America	8	1.25 [1.00, 1.56]	56%	
Europe	5	3.03 [2.08, 4.43]	77%	
Australia	1	1.95 [1.08, 3.53]	-	
**Effect estimate**				0.03
Adjusted for confounders	10	2.05 [1.39, 3.01]	91%	
Unadjusted for confounders	4	1.20 [0.89, 1.62]	41%	
**Measure of effect estimate**				1.00
ORs	7	1.81 [1.38, 2.36]	25%	
RRs	7	1.81 [1.10, 2.99]	95%	
**Sex**				0.74
Men	3	2.10 [1.00, 4.41]	91%	
Women	3	1.71 [0.64, 4.57]	0%	

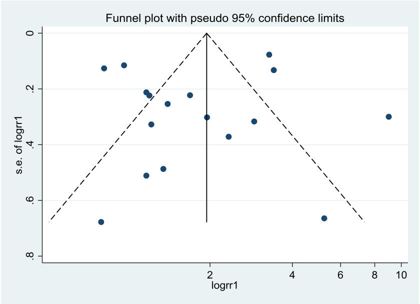

Figure E1: Funnel plot of alcohol vs no alcohol or lowest exposure to alcohol for studies presented the results as RRs.

PROSPERO International prospective register of systematic reviews

Review title and timescale

1 **Review title**

Give the working title of the review. This must be in English. Ideally it should state succinctly the interventions or exposures being reviewed and the associated health or social problem being addressed in the review.

Systematic review and meta-analysis of the effect of alcohol consumption on specific types of cancer and severe lung diseases

2 **Original language title**

For reviews in languages other than English, this field should be used to enter the title in the language of the review. This will be displayed together with the English language title.

3 **Anticipated or actual start date**

Give the date when the systematic review commenced, or is expected to commence.

01/12/2015

4 **Anticipated completion date**

Give the date by which the review is expected to be completed.

30/09/2018

5 **Stage of review at time of this submission**

Indicate the stage of progress of the review by ticking the relevant boxes. Reviews that have progressed beyond the point of completing data extraction at the time of initial registration are not eligible for inclusion in PROSPERO. This field should be updated when any amendments are made to a published record.

The review has not yet started √

Review stage	Started	Completed
Preliminary searches	Yes	No

Piloting of the study selection process	No	No
Formal screening of search results against eligibility criteria	No	No
Data extraction	No	No
Risk of bias (quality) assessment	No	No
Data analysis	No	No

Provide any other relevant information about the stage of the review here.

Review team details

6 Named contact

The named contact acts as the guarantor for the accuracy of the information presented in the register record.

Evangelia Simou

7 Named contact email

Enter the electronic mail address of the named contact.

msxes6@nottingham.ac.uk

8 Named contact address

Enter the full postal address for the named contact.

UK Centre for Tobacco and Alcohol Studies, Division of Epidemiology and Public Health, University of Nottingham, Clinical Sciences Building, Hucknall Road, Nottingham, NG5 1PB, UK

9 Named contact phone number

Enter the telephone number for the named contact, including international dialing code.

+44 (0) 115 82 31388

10 Organisational affiliation of the review

Full title of the organisational affiliations for this review, and website address if available. This field may be completed as 'None' if the review is not affiliated to any organisation.

The University of Nottingham

Website address:

http://nottingham.ac.uk/medicine/about/eph/index.aspx

11 Review team members and their organisational affiliations

Give the title, first name and last name of all members of the team working directly on the review. Give the organisational affiliations of each member of the review team.

Title	First name	Last name	Affiliation
Professor	John	Britton	Director, UK Centre for Tobacco & Alcohol Studies, Faculty of Medicine & Health Sciences,School of Medicine
Professor	Jo	Leonardi-Bee	UK Centre for Tobacco and Alcohol Studies, Faculty of Medicine & Health Sciences,School of Medicine,Univesrity of Nottingham

12 Funding sources/sponsors

Give details of the individuals, organizations, groups or other legal entities who take responsibility for initiating, managing, sponsoring and/or financing the review. Any unique identification numbers assigned to the review by the individuals or bodies listed should be included.

UK Centre for Tobacco & Alcohol Studies,(UKCTAS).

13 Conflicts of interest

List any conditions that could lead to actual or perceived undue influence on judgements concerning the main topic investigated in the review.

Are there any actual or potential conflicts of interest?

None known

14 Collaborators

Give the name, affiliation and role of any individuals or organisations who are working on the review but who are not listed as review team members.

Title	First name	Last name	Organisation details
Professor	Ian	Gilmore	

Review methods

15 Review question(s)

State the question(s) to be addressed / review objectives. Please complete a separate box for each question.

How does alcohol consumption impact on specific cancers and severe lung diseases on adults?

16 Searches

Give details of the sources to be searched, and any restrictions (e.g. language or publication period). The full search strategy is not required, but may be supplied as a link or attachment.

We will search the following electronic bibliographic databases: MEDLINE (via Ovid), EMBASE (via Ovid) and Web of Science. Studies published between 1985 and the date the searches are run will be sought. Emphasis will be given on the most recent studies. A 'search diary' will be kept giving details for the search strategy, including the names of the databases searched, the search terms used and the search results The search filter used by SIGN will be adopted to retrieve systematic reviews. Search terms for each health outcome will be developed from search strategies from relevant Cochrane Review groups. There will be no language restrictions.

17 URL to search strategy

If you have one, give the link to your search strategy here. Alternatively you can e-mail this to PROSPERO and we will store and link to it.

I give permission for this file to be made publicly available

Yes

18 Condition or domain being studied

Give a short description of the disease, condition or healthcare domain being studied. This could include health and wellbeing outcomes.

A full list of the outcomes being assessed are given under 'Primary outcomes' below.

19 Participants/population

Give summary criteria for the participants or populations being studied by the review. The preferred format includes details of both inclusion and exclusion criteria.

Inclusion: Adults aged 18 and over. Exclusion: Adults 18 years and older who do not consume alcohol.

20 Intervention(s), exposure(s)

Give full and clear descriptions of the nature of the interventions or the exposures to be reviewed

All studies which have assessed the effect of alcohol consumption defined as ever alcohol drinkers, ex-or former drinkers will be included. For the alcohol to include all drinking levels: light, moderate and heavy drinking, according to drinks/day or gr of ethanol/day), as defined in the included studies. Alternatively, for the drinking levels will be defined: 1 unit as 8 g or 10ml of ethanol, and light as ‹ 2 units per day; moderate as 2-3 units per day; heavy as >= 4 units per day, in accordance with standard recommended alcohol allowance guidance (UK). If a study does not report the alcohol consumption levels, a dichotomy of any alcohol consumption versus non- alcohol consumption will be used. We will exclude studies on special populations (alcoholics, patients HBV/HCV infected) and studies referred only on specific types of alcoholic beverages.

21 Comparator(s)/control

Where relevant, give details of the alternatives against which the main subject/topic of the review will be compared (e.g. another intervention or a non-exposed control group).

The comparison group will be adults who are not exposed to alcohol, or where drinking levels are considered within the included studies. Also, the comparison groups will be adults who are exposed to lower levels of alcohol consumption.

22 Types of study to be included

Give details of the study designs to be included in the review. If there are no restrictions on the types of study design eligible for inclusion, this should be stated.

We will include longitudinal or cohort studies which have assessed the effect of alcohol on the outcomes of interest. Where there is limited longitudinal evidence for particular outcomes, we will also include case control studies.

23 Context

Give summary details of the setting and other relevant characteristics which help define the inclusion or exclusion criteria.

24 Primary outcome(s)

Give the most important outcomes.

We will include all studies which assess the effect of alcohol on the incidence of the disease. Diagnosis of incidence of disease from death certificates will also be eligible for inclusion. We will also assess the effect of alcohol on specific cancer: Upper aerodigestive tract cancers (oral cavity, larynx,pharynx, esophagus), colorectum, liver, female breast, prostate, lung, bladder, pancreatic, endometrial, ovarian, skin cancer, renal cell, small intestine and leukemia. We will examine the association between alcohol and severe lung diseases: pneumonia, tuberculosis, acute respiratory distress syndrome, chronic obstructive pulmonary disease, asthma and sleep apnoea.

Give information on timing and effect measures, as appropriate.

We will include all relative effect measures, for example Hazard Ratios, Odds Ratios, risk Ratios.

25 Secondary outcomes

List any additional outcomes that will be addressed. If there are no secondary outcomes enter None.

None

Give information on timing and effect measures, as appropriate.

26 **Data extraction (selection and coding)**
Give the procedure for selecting studies for the review and extracting data, including the number of researchers involved and how discrepancies will be resolved. List the data to be extracted.
Two reviewers will examine independently both the titles and the abstracts that have identified by electronic search in order to select the relevant included articles. Then the full text of potentially eligible articles will be searched and read by the reviewers, checking each paper against the inclusion criteria. Any disagreements will be resolved through discussion with a third reviewer. Two reviewers will independently screen all the studies and abstracted the following information in a piloted and standard format: study design, time period, participants, exposures, study setting and outcomes related to cancer and severe lung diseases. Disagreements regarding eligibility will be resolved through discussion or with a third reviewer.

27 **Risk of bias (quality) assessment**
State whether and how risk of bias will be assessed, how the quality of individual studies will be assessed, and whether and how this will influence the planned synthesis.
Two reviewers will independently conduct the quality assessment and the risk of bias of the included studies using the Newcastle-Ottawa Scale for longitudinal and cohort studies and the Assessment of Multiple systematic Reviews (AMSTAR) Scale for systematic reviews. Disagreements between the review authors over the risk of bias in particular studies will be resolved by discussion, with involvement of a third review author where necessary.

28 **Strategy for data synthesis**
Give the planned general approach to be used, for example whether the data to be used will be aggregate or at the level of individual participants, and whether a quantitative or narrative (descriptive) synthesis is planned. Where appropriate a brief outline of analytic approach should be given.
We will conduct a meta-analysis to synthesize the data. We will extract specific effect measures for the association between alcohol consumption and the risk of the disease (cancer or lung infection). Risk estimates will be reported as odds ratios (OR), risk ratios (RR), hazard ratios (HR) or incidence rate ratios (IRR) with 95% confidence intervals (CI). We will use a random effect meta-analytic model to calculate summary estimates of similar studies. The I2 statistics will be used to evaluate heterogeneity and forest plots used for the graphic investigation of the heterogeneity. Also, funnel plots will be used to visually assess evidence of publication bias. We will also conduct sensitivity analysis by excluding each study at a time from the meta-analysis to assess the influence of individual studies on the pooled effect measure. All the statistical analyses will be carried out using the STATA software and Review Manager 5.3 version software.

29 **Analysis of subgroups or subsets**
Give any planned exploration of subgroups or subsets within the review. ‘None planned’ is a valid response if no subgroup analyses are planned.
If the necessary data are available,we will perform subgroup and meta-regression analyses to assess reasons for heterogeneity between the studies, based on the geographical area (studies conducted in Europe compared to the rest of the world), sex, and whether the results were adjusted for confounding.

Review general information

30 **Type and method of review**
Select the type of review and the review method from the drop down list.
Epidemiologic, Systematic review

31 **Language**
Select the language(s) in which the review is being written and will be made available, from the drop down list. Use the control key to select more than one language.
English
Will a summary/abstract be made available in English?
Yes

32 **Country**
Select the country in which the review is being carried out from the drop down list. For multi-national collaborations select all the countries involved. Use the control key to select more than one country.
England

33 **Other registration details**
Give the name of any organisation where the systematic review title or protocol is registered together with any unique identification number assigned. If extracted data will be stored and made available through a repository such as the Systematic Review Data Repository (SRDR), details and a link should be included here.

34 **Reference and/or URL for published protocol**
Give the citation for the published protocol, if there is one.
Give the link to the published protocol, if there is one. This may be to an external site or to a protocol deposited with CRD in pdf format.

I give permission for this file to be made publicly available

Yes

35 **Dissemination plans**

Give brief details of plans for communicating essential messages from the review to the appropriate audiences.

A qualitative evaluation will be conducted to gain an understanding of the public's beliefs of the harms of alcohol on health. All the findings from these reviews will be used to develop a comprehensive website, where the target audiences are the academic community, professionals and general public. This website will also be evaluated by the users to ensure it is understandable and accessible to the aforementioned targeted groups

Do you intend to publish the review on completion?

Yes

36 **Keywords**

Give words or phrases that best describe the review. (One word per box, create a new box for each term)

systematic review

meta-analysis

alcohol

cancer

lung diseases

37 **Details of any existing review of the same topic by the same authors**

Give details of earlier versions of the systematic review if an update of an existing review is being registered, including full bibliographic reference if possible.

38 **Current review status**

Review status should be updated when the review is completed and when it is published.

Ongoing

39 **Any additional information**

Provide any further information the review team consider relevant to the registration of the review.

40 **Details of final report/publication(s)**

This field should be left empty until details of the completed review are available.

Give the full citation for the final report or publication of the systematic review.

Give the URL where available.
