# Supplementary material for: Alcohol and the risk of pneumonia: a systematic review and meta-analysis
Source: BMJ Open. 2018 Aug 22;8(8):e022344. doi: 10.1136/bmjopen-2018-022344 (PMC6112384; doi:10.1136/bmjopen-2018-022344)
Supplement: Author's manuscript [file bmjopen-2018-022344.draft_revisions.pdf]

BMJ Open is committed to open peer review. As part of this commitment we make the peer review history of every article we publish publicly available.

When an article is published we post the peer reviewers' comments and the authors' responses online. We also post the versions of the paper that were used during peer review. These are the versions that the peer review comments apply to.

The versions of the paper that follow are the versions that were submitted during the peer review process. They are not the versions of record or the final published versions. They should not be cited or distributed as the published version of this manuscript.

BMJ Open is an open access journal and the full, final, typeset and author-corrected version of record of the manuscript is available on our site with no access controls, subscription charges or pay-per-view fees (http://bmjopen.bmj.com).

If you have any questions on BMJ Open's open peer review process please email
info.bmjopen@bmj.com

BMJ Open

Alcohol and the risk of pneumonia: A systematic review and meta-analysis

Journal:	*BMJ Open*
Manuscript ID	bmjopen-2018-022344
Article Type:	Research
Date Submitted by the Author:	14-Feb-2018
Complete List of Authors:	Simou, Evangelia; university of Nottingham, Epidemiology and Public Health/Clinical Science Building<br>Britton, John; University of Nottingham/ UK Centre for Tobacco and Alcohol Studies, Division of Epidemiology<br>Leonardi-Bee, Jo; Uni Nottingham, Epidemiology and Public Health/ Clinical Science Building
Keywords:	Respiratory infections < THORACIC MEDICINE, Epidemiology < THORACIC MEDICINE, Infectious disease/HIV < NEUROLOGY

SCHOLARONE™
Manuscripts

For peer review only

Alcohol and the risk of pneumonia: A systematic review and meta-analysis

Evangelia Simou[1], John Britton[1], Jo Leonardi-Bee[1]

[1]UK Centre for Tobacco and Alcohol Studies, Division of Epidemiology and Public Health, University of Nottingham, Hucknall Road, Nottingham NG5 1PB

Address for Correspondence: Evangelia Simou, PhD student, Division of Epidemiology and Public Health, University of Nottingham, Hucknall Road, Nottingham NG5 1PB, Tel: +44 115 82 31388 Fax: +44 115 82 31337, Email: Evangelia.simou@nottingham.ac.uk

John Britton, Professor of Epidemiology; Director, UK Centre for Tobacco & Alcohol Studies, Faculty of Medicine & Health Sciences

Jo Leonardi- Bee, Professor of Medical Statistics & Epidemiology, Faculty of Medicine & Health Sciences

Word Count: 3156

For peer review only

Abstract

Objective: A systematic review and meta-analysis to estimate the magnitude of the association between alcohol consumption and the risk of community acquired pneumonia (CAP) in adults was undertaken.
Design: Systematic review and meta-analysis
Methods: Comprehensive searches of Medline, EMBASE and Web of Science were carried out to identify comparative studies of the association between alcohol intake and CAP between 1985 and 2017. Reference lists were also screened. A random effects meta-analysis was used to estimate pooled effect sizes. A dose response meta-analysis was also performed.
Results: We found 17 papers eligible for inclusion in the review, of which 14 provided results which could be pooled. Meta-analysis of these 14 studies identified a 83% increased risk of CAP among people who consumed alcohol, or in higher amounts, relative to those who consumed no, or lower amounts of alcohol respectively (RR= 1.83, 95% CI: 1.30-2.57). There was substantial between-study heterogeneity, which was attributable in part to differences in study continent, adjustment for confounders, and pneumonia diagnosis (clinical vs death). Dose-response analysis found that for every 10-20 grams higher alcohol intake per day, there was 8% increase in the risk of CAP.
Conclusions: The findings suggest that high alcohol consumption increases the risk of CAP. Therefore, strengthening policies to reduce alcohol intake would be likely to reduce the incidence of CAP.

Key words: alcohol consumption; pneumonia; systematic review; meta-analysis; dose response analysis.

Strengths and limitations of this study

- This study represents a comprehensive review with no language restriction following the PRISMA and MOOSE guidelines.
- The heterogeneity was explored using subgroup analysis based on *a priori* defined factors.
- A dose response analysis identified a significant increase in CAP risk in relation to quantity of alcohol consumed.
- The grey literature was not searched in this review.

INTRODUCTION

Pneumonia is a major cause of global morbidity and mortality. In 2014 in the United States, pneumonia (including influenza) was the eighth leading cause of death (1) and according to World Health Organization, in 2015 pneumonia was responsible for 16% of all deaths in children aged under 5 years (2). Community acquired infections are the most common cause of pneumonia, and with an annual incidence in Europe and North America of between 5 to 11 cases per thousand adults (3), community acquired pneumonias (CAP) account for an annual total of 4 million deaths annually (4). Globally, Streptococcus pneumoniae is the most common pathogen causing CAP (5). The annual incidence of community-acquired pneumonia requiring hospitalization among US adults is 24.8 cases per 10,000 adults; with highest incidence especially in oldest people.(6). Patients with severe CAP admitted to European intensive care units have a mortality rate of 27% at six months (7).

For peer review only - http://bmjopen.bmj.com/site/about/guidelines.xhtml

Pneumonia is more common with increasing age (8, 9), among people who smoke (10-12) have a low body mass index (13) or have comorbidities including: other respiratory disease (12, 14), cardiovascular disease (14), stroke (14), dementia (11, 14) liver, or renal disease (14). Alcohol consumption is a potential risk factor for pneumonia. There are several possible mechanisms to explain the observation that alcohol consumption increases the risk of pneumonia, including the sedative properties of alcohol which can reduce oropharyngeal tone, leading to an increased risk of aspiration of microbes. Furthermore, high levels of alcohol intake can modify alveolar macrophage function, hence diminishing pulmonary defence against infection (15, 16). Also, high alcohol consumption is often associated with malnutrition (17) as it interacts with nutrient metabolism and utilization (18), resulting in the impairment of immunity and increases CAP risk.

To date however, evidence on the association between alcohol consumption and CAP is limited. A systematic review and meta-analysis published in 2010, using evidence published before August 2009, found a 6% increase in the risk of pneumonia per standard drink of 12 g of pure alcohol per day, but the number of studies reviewed (five) was small (19). However, there is an increase in the interest on this topic and also several studies have been published in the past nine years. For this reason we have carried out a systematic review and meta-analysis, to quantify the association between alcohol consumption and risk of CAP.

METHODS

The systematic review and meta-analysis was carried out in adherence with PRISMA (20) and MOOSE (21) guidelines. The protocol was published in the National Institute for Health Research International prospective register of systematic reviews (PROSPERO) under the registration number: 42015029910.

Inclusion criteria

The PICO criteria were used for the eligibility of the articles based on type of study design, type of population, type of exposure and outcome. We included all comparative study designs (longitudinal, cohort, case-control, and cross sectional) assessing the association between alcohol intake and the risk of CAP in generally representative adult populations (>=18 years), and therefore excluded studies of selected populations such as people with HIV, Hepatitis B, or C virus infection; and those with hospital-acquired pneumonia. Where possible, we also analysed the association between alcohol consumption and the occurrence of pneumonia due to specific organisms (for example, Streptococcus pneumonia).

Exposure ascertainment

Alcohol consumption defined either by self-report (interview or questionnaire) or using medical records. Also, alcohol use corresponded to drinking levels (low, moderate, heavy, and alcoholism) or to frequency measures (grams/units/drinks per day/week).

Outcome ascertainment

Community acquired pneumonia diagnosis based: on a clinical diagnosis (chest x-ray, blood test), physician diagnosis and medical records including ICD codes or self-report.

Search strategy

Comprehensive search strategies were applied to the Medline (via Ovid), EMBASE (via Ovid), and Web of Science databases for the period from December 1985 to December 2017. We

For peer review only - http://bmjopen.bmj.com/site/about/guidelines.xhtml

used search filters for observational study designs (22) and search terms for both outcome and exposure developed from relevant Cochrane Review groups (23). The Medline search filters were the following: exp Alcohol-Related Disorders/ OR Alcohol Drinking/OR (alcohol adj3 (drink$ or intoxicat$ or use$ or abus$ or misus$ or risk$ or consum$ or withdraw$ or detox$ or treat$ or therap$ or excess$ or reduc$ or cessation or intervention$)).tw. OR (drink$ adj3 (excess or heavy or heavily or harm or harmful or hazard$ or binge or problem$)).tw. OR alcoholic$.tw. AND [exp Respiratory Tract Infections/ OR acute respiratory infection*.tw. OR lower respiratory infection*.tw. OR lower respiratory tract infection*.tw. OR exp Pneumonia/OR (pneumon* or bronchopneumon* or pleuropneumon*).tw. OR exp Bronchitis/ OR (bronchit* or bronchiolit*).tw]. The full search strategy is presented (see Table E1 in the online data supplement). Reference lists of included studies were also screened in order to identify further potentially eligible studies. No language limitation was imposed and where necessary papers were translated into English. Where there was more than one report of findings from the same population (for example an abstract and then a full paper), the most recently published version of the study was used. Screening of titles and abstracts, as well as the full text, was conducted independently by two reviewers (ES and JL-B). Any disagreements were resolved thought discussion, or with the help of the third reviewer (JB).

Data extraction

Two reviewers (ES and JL-B) independently extracted data using a previously piloted form, which included the following information: author, year, study design, definitions of exposure (alcohol) and outcome (community acquired pneumonia), geographic location, reference population, and adjustment for confounders.

For categorical measures of alcohol drinking, where possible we compared any alcohol consumption with no alcohol consumption (reference group), or else used the lowest exposed category as the reference group. Also, in the main analysis, categorical measures of alcohol consumption were further defined as levels of consumption: light, moderate, heavy, and alcoholism. Grams of daily alcohol consumption were used as a standard measure, defining: one drink as 0·6 ounces, 14·0 grams, or 1·2 tablespoons of pure alcohol (24). According to CDC guidelines, we defined heavy drinking as a weekly consumption of 15, or more drinks for men, and 8 or more drinks for women; binge drinking as five, or more drinks during a single occasion for men, or four or more for women; and excessive drinking as the presence of either binge or heavy drinking (24). Moderate alcohol drinking defined as the daily consumption of up to one drink for women and two drinks for men (25).

Quality assessment

Two authors (ES and JL-B) independently assessed the methodological quality of the included studies using the Newcastle-Ottawa Quality Scale (26).The maximum score for cohort and case control studies was nine and for cross sectional studies seven. Discrepancies were resolved through discussion and consensus. A score of 6, or more was deemed to be of high quality. We did not attempt to assess the methodological quality for studies published only in abstract form.

Statistical analysis

Relative measures of risk were extracted as odds ratios (OR), relative risks (RR) or hazard ratios (HR) with 95% confidence intervals. Where available, we used measures of risk

For peer review only - http://bmjopen.bmj.com/site/about/guidelines.xhtml

adjusted for smoking and socioeconomic status and extracted results separately for men and women. Where raw data were extracted from studies, we estimated ORs for case control studies and RRs for longitudinal, cohort and cross sectional studies. Where exposure to alcohol was reported using quantiles, or categories, we extracted adjusted effect measures relating to a comparison of the highest to the lowest exposure group. We pooled ORs and RRs together to estimate pooled RRs where the outcome measure was not assumed to be common; however HRs were not pooled with other effect measures. Meta-analysis was conducted, based on the DerSimonian and Laird's random effects model, to pool the results from the individual studies.

Heterogeneity between studies was quantified using I^2 statistics (27); and explored using subgroup analyses according to study quality, study design, adjustment for confounders, alcohol reference group (no alcohol vs lowest exposed category), CAP diagnosis (clinical diagnosis vs death records) and geographical location (Low and Middle Income Countries versus High Income Countries). Funnel plots were used as a visual aid to detect publication bias and where data for at least ten studies were available we formally assessed publication bias using Egger's asymmetry test. We performed all analyses using Stata (Version 14) and Review Manager (Version 5·3). All p-values <0.05 were deemed to represent statistical significance.

Dose response assessment

To assess the evidence for causality, we applied a modified version of Hill's criteria to assess causation (28) on strength of association, consistency, temporality, biological gradient and plausibility. To assess the biological gradient criterion we performed a random effects dose-response meta-analysis (29, 30), where we assumed a linear dose- response relation and allowed for study level correlations across the categories of quantities of alcohol. The dose-response relation between alcohol consumption and CAP was analysed using the subgroup of studies including at least three different categories of exposure, standardized for analysis to grams per day, and where appropriate using the midpoint of categories defined by ranges of intake. If the highest exposure category was open-ended, we took the highest category midpoint to be the lower bound plus 1.2 times the lower boundary (31). When available we included results for men and women separately.

Separate dose-response meta- analyses were conducted for cohort/ longitudinal and case control/ cross sectional studies. Dose categories relating to quantities of alcohol were created to equate to 10-20 grams of pure alcohol per day (approximately one drink per day); where studies reported categories which contained the same dose ranges we collapsed these into a single dose category through estimating a pooled effect estimates based on a fixed effect meta- analysis model. Where necessary, effect estimates and 95% CI were back calculated from floated to conventional confidence intervals to enable comparisons to be made to the reference group (non- drinkers or the lowest exposed category (32).

RESULTS

The searches identified a total of 4589 studies published between December 1985 and December 2017, of which 17 were eligible for inclusion in the systematic review (Figure 1). The characteristics of the 17 included studies are presented in Table 1. A total population of 287,184 people was included in our review. Seven studies used a cohort, or longitudinal design (10, 33-38), nine used a case control design (11, 39-46) and one used a cross sectional design(47). Eight studies were conducted in America (10, 11, 37, 38, 44-47); five in Europe

For peer review only - http://bmjopen.bmj.com/site/about/guidelines.xhtml

(35, 39, 41-43), two in Asia (33, 34) and two in Australia (36, 40). Three studies reported separate estimates of the association between alcohol and CAP for men and women (10, 39, 42), and 12 studies reported effect estimates adjusted for confounders (10, 33, 34, 37, 39, 41-47).

The majority of studies assessed alcohol consumption by self-report, based either on a standardized questionnaire, or on an interview while five studies used reported intake data from medical records (11, 35, 38, 44, 45). The reference group for nine studies comprised people who never consumed alcohol(10, 33, 34, 36, 40, 42, 43, 45, 46); whereas the reference group for the remaining eight studies comprised people who consumed the lowest quantity of alcohol.

Seven studies ascertained CAP using a clinical diagnosis; and five of these used chest x-ray radiography (40-43, 46). A further seven studies ascertained CAP using ICD codes (33, 34, 36, 38, 39, 44) and medical records (44) and two studies used self-report interview (37, 47). The remaining study ascertained CAP via physician diagnosis using medical records (10).

The methodological quality of the included studies ranged from five to eight, with a median score of six. Ten studies were deemed to be of high quality (>6) (10, 33, 35-37, 39, 41, 43-45); whereas lower scores tended to arise from failure to adjust for confounders, or using self-reported methods to ascertain alcohol consumption (Table 2).

For peer review only

For peer review only - http://bmjopen.bmj.com/site/about/guidelines.xhtml

Table 1. Characteristics of the included studies

* Crude analysis reported

Study & Year	Study design	Geographical location	Alcohol ascertainment	Alcohol definition	CAP ascertainment	Confounders adjusted
Almirall 1999 (43)	Case control	Europe/Spain	Self-report/questionnaire	Quartiles of alcohol intake >35·3 versus 0 (grams/day)	Clinically suspected and chest radiography	Age, sex, municipality
Almirall 2008 (42)	Case control	Europe/Spain	Self-report /questionnaire	Quartiles of alcohol intake(grams/day) Men: >80 versus 0 Women: >40 versus 0	Clinically suspected and chest radiography	Age, sex, primary care practice
Baik 2001 (10)	Cohort	America/US	Self-report/questionnaire	Men: >30 versus never Women: >30 versus never (Grams/day)	Physician diagnosis/Medical records	Age, smoking status, BMI, quintile of metabolic equivalent
Breitling 2016 (37)	Cohort	America/US	Self-report/questionnaire	Quartiles of alcohol intake Men: >20 versus ≤ 20 Women: >10 versus 0 (grams/day)	Self-report questionnaire	Age, sex, smoking, BMI, diabetes mellitus, stroke, congestive heart failure, cancer
Clough 2003 (40)	Case control	Australia	Self-report/interview	Alcohol yes versus alcohol no	Clinically suspected/ X-ray findings	-*
Fernandez-Sola 1995 (41)	Case control	Europe/Spain	Self-report/ Interview & questionnaire	High intake (men: >100g, women:>800g) versus low intake (grams/day 2 years before submission)	Clinically suspected/ Chest X-ray	Liver cirrhosis, smoking, COPD, diabetes, heart failure, malnutrition
Innoue 2007 (34)	Cohort	Asia/Japan	Self-report/questionnaire	Current versus never drinking	Mortality ICD codes	Age and history of diabetes mellitus
Jackson 2009 (44)	Case control	America/US	Medical records	Current alcoholism vs no alcoholism	ICD9 codes	Age, sex, pneumonia-free person-time
Koivula 1994(35)	Cohort	Europe/Finland	Medical records	Alcoholism vs no alcoholism	Medical records	Age, sex, chronic conditions
Lipsky 1986 (11)	Case control	America/US	Medical records	Heavy versus moderate (drinks/day)	Clinically suspected	-*
Loeb 2009 (46)	Case control	America/US	Self-report /questionnaire	Alcohol yes (previous 12 months) versus alcohol no(grams/month)	Clinically suspected and chest radiography	Multivitamins, smoking, history of gas and fumes exposure
Phung 2013(36)	Cohort	Australia	Self-report/questionnaire	Alcohol yes versus alcohol no	Hospital records -ICD codes	-*
Quraishi 2013(47)	Cross sectional	America/US	Self-report/interview	alcohol consumption (≤30 versus >30 drinks per month)	Self-report interview	-*
Shen 2013 (33)	Cohort	Asia/China	Self-report/interview	Excessive versus never drinkers (units/week)	Mortality ICD codes	Age, Sex, education, housing, monthly expenditure, smoking, BMI, exercise, health status
Watt 2007 (45)	Case control	America/US	Medical records	Alcoholism/ alcohol use versus no use of alcohol	Clinically suspected Pneumococcal isolation in patient from sterile body fluid	Smoking, BMI, electricity/ indoor plumbing in home, living with unvaccinated child, unemployed, wood/coal, smoke
Yende 2013 (38)	Cohort	America/US	Medical records	Alcohol abuse vs no alcohol abuse	ICD-9 codes	-*
Zaridze 2009 (39)	Case control	Europe/Russia	Self-report interview	≥3bottles(per week) versus <=0·5 bottles of vodka	ICD codes Death records	Age, city, and smoking

For peer review only - http://bmjopen.bmj.com/site/about/guidelines.xhtml

Table 2. Quality assessment- Newcastle Ottawa scale

Study, Year	Stars number			
	Selection†	Comparability‡	Exposure§	Overall
Almirall 1999 (43)	4	1	1	6/9
Almirall 2008 (42)	3	1	1	5/9
Baik 2001 (10)	4	2	2	8/9
Breitling 2016	3	2	2	5/9
Clough 2003 (40)	4	0	1	5/9
Fernandez-Sola 1995 (41)	3	2	1	6/9
Innoue 2007 (34)	3	1	1	5/9
Jackson 2009 (44)	4	1	1	6/9
Koivula 1994(35)	4	1	3	8/9
Lipsky 1986 (11)	3	0	2	5/9
Loeb 2009 (46)	2	2	1	5/9
Phung 2013(36)	3	0	3	6/9
Quraishi 2013(47)	1	0	1	2/6
Shen 2013 (33)	3	2	3	8/9
Watt 2007 (45)	4	2	1	7/9
Yende 2013	4	0	2	6/9
Zaridze 2009 (39)	3	2	1	6/9

Meta-analysis findings

Fourteen of the 17 included studies provided data from which pooled relative risks could be estimated, and a pooled analysis of these studies found the risk of CAP to be significantly increased in people who consumed alcohol at all, or in higher amounts, relative to those who consumed no, or lower amounts of alcohol respectively (pooled RR= 1.83, 95% CI 1.30 to 2.57, I^2= 91%, Figure 2). There was evidence of publication bias detected visually via a funnel plot, and statistically via Egger's asymmetry test (P = 0.596).

Subgroup analyses exploring the reason for heterogeneity in the meta-analysis of these 14 studies are presented in the Supplementary material (see Table E2). Heterogeneity was not explained by study design (case control, longitudinal/cohort, cross sectional; p for subgroup differences=0.07), methodological quality (high versus low; p=0.09) or gender (male versus female; p=0.74). However, significant differences were found according to adjustment for confounders (adjusted versus unadjusted; p=0.03), continent of study (America, Europe, Australia; p=0.0003), and ascertainment of CAP (clinical diagnosis vs death records; p=0.002). Additionally, no significant differences were found by the definition of the reference group for alcohol consumption (p=0.39; Figure 2).

A sensitivity analysis restricted to the six studies which provided smoking-adjusted estimates found a larger magnitude of effect compared to the main analysis (pooled RR= 2.01, 95% CI 1.25 to 3.23, I^2=93%, 6 studies). Similarly the studies provided age-adjusted effect estimated found a risk of 1.90 (pooled RR= 1.90, 95% CI 1.20 to 3.02, I^2=93%, 7 studies).

The remaining three studies presented effect estimates as hazard ratios (33, 34, 36), and a pooled analysis of these studies estimated a hazard ratio for CAP in relation to alcohol consumption of 0.90 (95%CI: 0.79 to 1.03, I^2=0).

Two studies assessing the effect of alcohol on pneumococcal disease specific strains of pneumonia were identified (11, 45) . A pooled analysis of these studies found that there was

For peer review only - http://bmjopen.bmj.com/site/about/guidelines.xhtml

more than a doubling of risk of Streptococcus CAP in people who consumed alcohol (RR= 2·16, 95% CI 1·05 to 4·48, I^2=42%)

Biological gradient meta-analysis

Five of the included studies provided data enabling a dose-response meta-analysis (10, 39, 40, 42, 43); of which: one used a cohort design (data reported separately for men and women) and four were case-control studies. A pooled analysis of the dose-response data from the cohort study found no significant gradient in the quantity of alcohol associated with the risk of CAP (p for trend=0·136). In contrast, the pooled analysis of the dose-response data from the four case control studies indicated that there was a significant gradient in the quantity of alcohol associated with a 8% increase in the risk of CAP for every 10-20 grams of pure alcohol consumed per day (equivalent to 1 drinks/day) (pooled RR= 1·08, 95% CI 1·06 to 1·09; p<0·0001; Figure 3)

DISCUSSION

Alcohol consumption is a recognised and avoidable risk factor for a range of diseases and injuries, including neuropsychiatric conditions, gastrointestinal and cardiovascular disease, cancer, suicide, violence and tuberculosis (48). To date however the association between alcohol consumption and pneumonia risk has attracted relatively little attention.

Summary of the findings

This meta-analysis of studies published over the past 30 years- demonstrates a clear and statistically significant relation between alcohol consumption and the risk of community acquired pneumonia. The effect was strong, with a 1.8 fold increase in risk among those with relatively high intakes of alcohol and significantly related to level of intake, with no evidence of publication bias.

Strengths and limitations

This study represents a comprehensive review of the global literature with no language restriction, making this analysis the most complete to date, and our findings likely to be generalizable. There was significant heterogeneity between the studies in our analysis, but our subgroup analyses indicate that this arose primarily from the continent in which the study was carried out (America, Europe, Australia); adjustment for confounders; and the ascertainment of CAP (death vs clinical diagnosis). Misclassification bias arising from inclusion of non-drinkers in the lowest category of alcohol intake in some studies can be another possible limitation in our review, but will resulted in a more conservative estimate of effect. A dose response relationship was identified. However the included studies did not reported dose response relations separately for men and women, so we are unable to carry out a comparative analysis.

Comparison with other studies

Our findings extend those of an earlier review and meta-analysis, carried out in 2010 (19). Another review focussed on risk factors for invasive pneumococcal diseases, indicated an

For peer review only - http://bmjopen.bmj.com/site/about/guidelines.xhtml

elevated risk for invasive pneumococcal disease due to alcohol consumption in six of the four studies included in the meta-analysis model (49). Likewise, another recent meta-analysis indicated an elevated risk for invasive pneumococcal disease due to alcohol consumption in six of the four studies included in the meta-analysis model (50). Similarly our separate meta-analysis focused on pneumococcal infections including two of these studies, due to our eligibility criteria, showed an elevated risk for pneumococcal acquisition.

A previous systematic review and meta-analysis found that people with a daily alcohol consumption of either 24, 60, and 120 grams have a 12 % ,33% and 76 % increased risk of CAP respectively (19). Our dose response analysis generated a slightly less strong effect, of an 8% increase in risk per 10-20 grams of (pure) alcohol consumed per day.

A general systematic review published by Almirall et al in 2017 (51) focused on risk factors of community acquired pneumonia, but provided only a narrative summary of findings and stating that no definite conclusion could be drawn. In contrast, our review found evidence of a doubling in the risk of CAP in people who consumed alcohol. Furthermore, our demonstration of a significant exposure-response association increases the likelihood, given the strength of the observed association and its consistency across a range of subgroups, that the observed association is causal. Further evidence of causality arises from studies demonstrating that alcohol consumption impairs alveolar macrophages and increases carriage of pneumonia pathogens (15, 16, 52).

Conclusion

Our findings thus provide clear evidence that relatively high intakes of alcohol increase the risk of pneumonia and therefore that measures to reduce alcohol intake are likely to reduce mortality and morbidity from community-acquired pneumonia.

Contributors

ES, JB and JL-B designed the study and wrote the protocol. ES wrote the search strategy and undertook the literature searches, and wrote the draft of the manuscript. ES and JLB undertook study screening, data extraction, and quality assessment. ES undertook all data analysis, supervised by JL-B. All authors contributed to the interpretation of the findings. JB and JLB provided critical revisions to the article, and all authors approved the final version of the article to be published. ES acts as guarantor of the manuscript.

Conflicts of interest

None declared

Acknowledgments

The authors thank Erica Brasil, Magdalena Opazo-Breton and Yue Huang from the University of Nottingham for their help in translations

Funding: This work was supported by the Medical Research Council [grant number MR/K023195/1]; the UK Centre for Tobacco and Alcohol Studies (http://www.ukctas.net); and the British Heart Foundation, Cancer Research UK, the Economic and Social Research Council, and the National Institute of Health Research, under the auspices of the UK Clinical Research Collaboration, is gratefully acknowledged.

Ethics approval: Ethics approval was not required for this work

Data sharing: No additional data are available.

For peer review only - http://bmjopen.bmj.com/site/about/guidelines.xhtml

References

1. Murphy SL, Kochanek KD, Xu J, Arias E. Mortality in the United States, 2014. NCHS data brief. 2015;229:1-8.
2. World Health Organization, Pneumonia Fact Sheet;World Health Organization Report 2016, WHO: Geneva, Switzerland. 2016.
3. Rozenbaum M, Pechlivanoglou P, Van Der Werf T, Lo-Ten-Foe J, Postma M, Hak E. The role of Streptococcus pneumoniae in community-acquired pneumonia among adults in Europe: a meta-analysis. Eur J Clin Microbiol Infect Dis. 2013;32(3):305-16.
4. Ruuskanen O, Lahti E, Jennings LC, Murdoch DR. Viral pneumonia. The Lancet. 2011;377(9773):1264-75.
5. Woodhead M. Community-acquired pneumonia in Europe: causative pathogens and resistance patterns. Eur Respir J. 2002;20(36 suppl):20s-7s.
6. Jain S, Self WH, Wunderink RG, Fakhran S, Balk R, Bramley AM, et al. Community-acquired pneumonia requiring hospitalization among US adults. N Engl J Med. 2015;373(5):415-27.
7. Walden AP, Clarke GM, McKechnie S, Hutton P, Gordon AC, Rello J, et al. Patients with community acquired pneumonia admitted to European intensive care units: an epidemiological survey of the GenOSept cohort. Critical care. 2014;18(2):R58.
8. European Commission. Health statistics, Atlas on mortality in the European Union, Luxembourg: Office for Official Publications of the European Communities. 2008.
9. Jain S, Williams DJ, Arnold SR, Ampofo K, Bramley AM, Reed C, et al. Community-acquired pneumonia requiring hospitalization among US children. N Engl J Med. 2015;372(9):835-45.
10. Baik I, Curhan GC, Rimm EB, Bendich A, Willett WC, Fawzi WW. A prospective study of age and lifestyle factors in relation to community-acquired pneumonia in US men and women. Arch Intern Med. 2000;160(20):3082-8.
11. Lipsky BA, Boyko EJ, Inui TS, Koepsell TD. Risk factors for acquiring pneumococcal infections. Arch Intern Med. 1986;146(11):2179-85.
12. Kohlhammer Y, Schwartz M, Raspe H, Schäfer T. Risk factors for community acquired pneumonia (CAP). A systematic review. Deutsche medizinische Wochenschrift (1946). 2005;130(8):381-6.
13. Simet SM, Sisson JH. Alcohol's effects on lung health and immunity. Alcohol research: current reviews. 2015;37(2):199.
14. Vinogradova Y, Hippisley-Cox J, Coupland C. Identification of new risk factors for pneumonia: population-based case-control study. Br J Gen Pract. 2009;59(567):e329-e38.
15. Mehta AJ, Guidot DM. Pathophysiology Review Series: Alcohol Abuse, the Alveolar Macrophage, and Pneumonia. The American journal of the medical sciences. 2012;343(3):244.
16. Kershaw CD, Guidot DM. Alcoholic lung disease. Alcohol Research & Health. 2008;31(1):66-76.
17. Moss M. Epidemiology of sepsis: race, sex, and chronic alcohol abuse. Clin Infect Dis. 2005;41(Supplement_7):S490-S7.
18. Marsano L. Alcohol and malnutrition. Alcohol Research and Health. 1993;17(4):284.
19. Samokhvalov A, Irving H, Rehm J. Alcohol consumption as a risk factor for pneumonia: a systematic review and meta-analysis. Epidemiology and infection. 2010;138(12):1789-95.
20. Moher D, Liberati A, Tetzlaff J, Altman DG. Preferred reporting items for systematic reviews and meta-analyses: the PRISMA statement. Ann Intern Med. 2009;151(4):264-9.
21. Stroup DF, Berlin JA, Morton SC, Olkin I, Williamson GD, Rennie D, et al. Meta-analysis of observational studies in epidemiology: a proposal for reporting. Journal of American Medical Association. 2000;283(15):2008-12.
22. SIGN. Search Filters, Obseravtional studies, Available from: http://www.sign.ac.uk/methodology/filters.html#obs (accessed 4 December 2015).
23. Cochrane Library. Available from: http://www.cochranelibrary.com/ (accessed 4 December 2015).

For peer review only - http://bmjopen.bmj.com/site/about/guidelines.xhtml

24. Centers for Disease Control and Prevention (CDC). Alcohol & Public Health, Fact Sheets - Alcohol Use and Your Health, Available from: http://www.cdc.gov/alcohol/fact-sheets/alcohol-use.htm (accessed 10 December 2015).
25. U.S. Department of Health and Human Services and U.S. Department of Agriculture. 2015 – 2020. Dietary Guidelines for Americans. 8th Edition, Washington, DC; 2015.
26. Wells G, Shea B, O'connell D, Peterson J, Welch V, Losos M, et al. The Newcastle-Ottawa Scale (NOS) for assessing the quality of nonrandomised studies in meta-analyses http://www.ohri.ca/programs/clinical_epidemiology/oxford.asp (accessed 10 February 2016).
27. Higgins J, Thompson SG. Quantifying heterogeneity in a meta-analysis. Statistics in Medicine. 2002;21(11):1539-58.
28. Hill AB. The environment and disease: association or causation? : SAGE Publications; 1965.
29. Greenland S, Longnecker MP. Methods for trend estimation from summarized dose-response data, with applications to meta-analysis. Am J Epidemiol. 1992;135(11):1301-9.
30. Orsini N, Bellocco R, Greenland S. Generalized least squares for trend estimation of summarized dose-response data. Stata Journal. 2006;6(1):40.
31. Berlin JA, Longnecker MP, Greenland S. Meta-analysis of epidemiologic dose-response data. Epidemiology. 1993;4(3):218-28.
32. Orsini N. From floated to conventional confidence intervals for the relative risks based on published dose–response data. Computer methods and programs in biomedicine. 2010;98(1):90-3.
33. Shen C, Ni MY, Schooling CM, Chan WM, Lee SY, Lam TH. Alcohol use and death from respiratory disease in a prospective Chinese elderly cohort study in Hong Kong. Prev Med. 2013;57(6):819-23.
34. Inoue Y, Koizumi A, Wada Y, Iso H, Watanabe Y, Date C, et al. Risk and protective factors related to mortality from pneumonia among middleaged and elderly community residents: the JACC Study. J Epidemiol. 2007;17(6):194-202.
35. Koivula I, Sten M, Makela PH. Risk factors for pneumonia in the elderly. The American journal of medicine. 1994;96(4):313-20.
36. Phung DT, Wang Z. Risk of pneumonia in relation to body mass index in Australian Aboriginal people. Epidemiol Infect. 2013;141(12):2497-502.
37. Breitling LP, Saum K-U, Schöttker B, Holleczek B, Herth FJ, Brenner H. Pneumonia in the Noninstitutionalized Older Population: A prospective observational study over 11 years. Deutsches Ärzteblatt International. 2016;113(37):607.
38. Yende S, Alvarez K, Loehr L, Folsom AR, Newman AB, Weissfeld LA, et al. Epidemiology and long-term clinical and biologic risk factors for pneumonia in community-dwelling older Americans: analysis of three cohorts. Chest. 2013;144(3):1008-17.
39. Zaridze D, Brennan P, Boreham J, Boroda A, Karpov R, Lazarev A, et al. Alcohol and cause-specific mortality in Russia: a retrospective case-control study of 48 557 adult deaths. Lancet. 2009;373(9682):2201-14.
40. Clough AR, Wang Z, Bailie RS, Burns CB, Currie BJ. Case-control study of the association between kava use and pneumonia in eastern Arnhem and Aboriginal communities (Northern Territory, Australia). Epidemiol Infect. 2003;131(1):627-35.
41. Fernandezsola J, Junque A, Estruch R, Monforte R, Torres A, Urbanomarquez A. HIGH ALCOHOL INTAKE AS A RISK AND PROGNOSTIC FACTOR FOR COMMUNITY-ACQUIRED PNEUMONIA. Arch Intern Med. 1995;155(15):1649-54.
42. Almirall J, Bolibar I, Serra-Prat M, Roig J, Hospital I, Carandell E, et al. New evidence of risk factors for community-acquired pneumonia: a population-based study. Eur Respir J. 2008;31(6):1274-84.
43. J. Almirall IB, X. BalanzoÂ, C.A. GonzaÂ lez. Risk factors for community-acquired pneumonia in adults:

a population-based case±control study. Eur Respir J. 1999;13:349-55.

For peer review only - http://bmjopen.bmj.com/site/about/guidelines.xhtml

44. Jackson ML, Nelson JC, Jackson LA. Risk Factors for Community-Acquired Pneumonia in Immunocompetent Seniors. J Am Geriatr Soc. 2009;57(5):882-8.
45. Watt JP, O'Brien KL, Benin AL, McCoy SI, Donaldson CM, Reid R, et al. Risk factors for invasive pneumococcal disease among Navajo adults. Am J Epidemiol. 2007;166(9):1080-7.
46. Loeb M, Neupane B, Walter SD, Hanning R, Carusone SC, Lewis D, et al. Environmental Risk Factors for Community-Acquired Pneumonia Hospitalization in Older Adults. J Am Geriatr Soc. 2009;57(6):1036-40.
47. Quraishi SA, Bittner EA, Christopher KB, Camargo Jr CA. Vitamin D status and community-acquired pneumonia: results from the third National Health and Nutrition Examination Survey. PLoS ONE. 2013;8(11):e81120.
48. World Health Organization. Global status report on alcohol and health 2014.
49. Boé DM VR, Burnham EL, Moss M. . Alcohol abuse and pulmonary disease. . Journal of leukocyte biology. 2009 Nov 1;86(5):1097-104.
50. Cruickshank HC, Jefferies JM, Clarke SC. Lifestyle risk factors for invasive pneumococcal disease: a systematic review. BMJ Open. 2014;4(6):e005224.
51. Almirall J, Serra-Prat M, Bolíbar I, Balasso V. Risk Factors for Community-Acquired Pneumonia in Adults: A Systematic Review of Observational Studies. Respiration. 2017.
52. Dao TT, Liebenthal D, Tran TK, Ngoc Thi Vu B, Ngoc Thi Nguyen D, Thi Tran HK, et al. Klebsiella pneumoniae oropharyngeal carriage in rural and urban Vietnam and the effect of alcohol consumption. PLoS ONE. 2014;9(3):e91999.

Table of contents

Table 1. Characteristics of the included studies... 6
Table 2. Quality assessment- Newcastle Ottawa scale 7

Figure legend

Figure 1. Study selection...
Figure 2. Forest plot of alcohol consumption and risk of CAP; subgroup analysis based on reference group(never drinking versus lowest drinking category) ....
Figure 3. Linear dose response meta-analysis for the association between alcohol intake categories (grams/day) and the risk of CAP ....

For peer review only

For peer review only - http://bmjopen.bmj.com/site/about/guidelines.xhtml

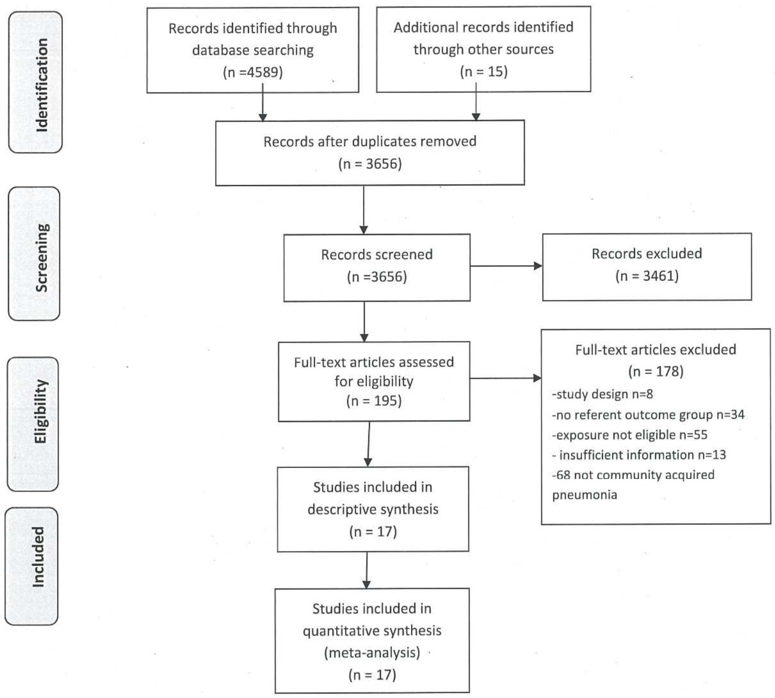

Figure 1. Study selection

209x298mm (300 x 300 DPI)

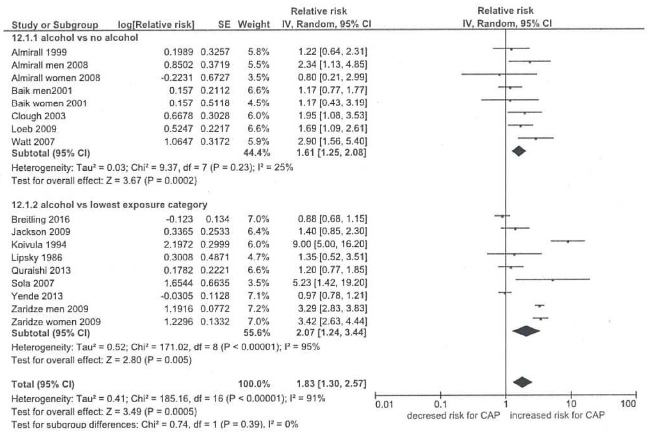

Figure 2. Forest plot of alcohol consumption and risk of CAP; subgroup analysis based on reference group(never drinking versus lowest drinking category)

209x298mm (300 x 300 DPI)

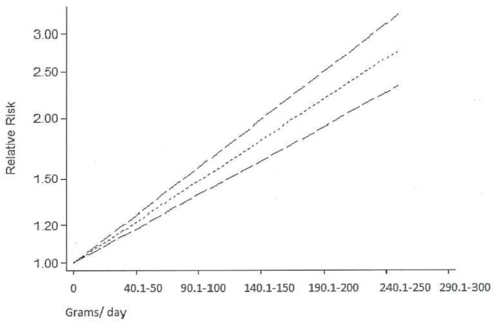

Figure 3. Linear dose response meta-analysis for the association between alcohol intake categories (grams/day) and the risk of CAP

209x298mm (300 x 300 DPI)

Alcohol and the risk of pneumonia: A systematic review and meta-analysis

Evangelia Simou, John Britton, Jo Leonardi-Bee

Online Data Supplement

**Table E1: Medline (via Ovid) and EMBASE (via Ovid) search terms for primary studies**
**Medline via Ovid search terms**
1. Epidemiologic studies/
2. Exp case control studies/
3. Exp cohort studies/
4. Case control.tw.
5. (cohort adj (study or studies)).tw.
6. Cohort analy$.tw.
7. (Follow up adj (study or studies)).tw.
8. (observational adj (study or studies)).tw.
9. Longitudinal.tw.
10. Retrospective.tw.
11. Cross sectional.tw.
12. Cross-sectional studies/
13. Or/1-12
14. exp Alcohol-Related Disorders/
15. Alcohol Drinking/
16. (alcohol adj3 (drink$ or intoxicat$ or use$ or abus$ or misus$ or risk$ or consum$ or withdraw$ or detox$ or treat$ or therap$ or excess$ or reduc$ or cessation or intervention$)).tw.
17. (drink$ adj3 (excess or heavy or heavily or harm or harmful or hazard$ or binge or problem$)).tw.
18. alcoholic$.tw.
19. 14 or 15 or 16 or 17 or 18
20. exp Respiratory Tract Infections/
21. acute respiratory infection*.tw.
22. lower respiratory infection*.tw.
23. lower respiratory tract infection*.tw.
24. exp Pneumonia/
25. (pneumon* or bronchopneumon* or pleuropneumon*).tw.
26. exp Bronchitis/
27. (bronchit* or bronchiolit*).tw.
28. 20 or 21 or 22 or 23 or 24 or 25 or 26 or 27
29. 13 and 19 and 28
**Embase via Ovid search terms**
1. Clinical study/
2. Case control study
3. Family study/
4. Longitudinal study/
5. Retrospective study/
6. Prospective study/
7. Randomized controlled trials/
8. 6 not 7
9. Cohort analysis/
10. (Cohort adj (study or studies)).mp.
11. (Case control adj (study or studies)).tw.
12. (follow up adj (study or studies)).tw.
13. (observational adj (study or studies)).tw.
14. (epidemiologic$ adj (study or studies)).tw.
15. (cross sectional adj (study or studies)).tw.
16. Or/1-5,8-15
17. substance-related disorders/
18. ((drug or substance) adj (Addict$ or abus$ or dependen$)).mp
19. (intoxicat$ or abstinen$ or withdrawal$).mp.
20. (excessive$ adj use$).mp.
21. (use$ adj disorder$).mp.

For peer review only

22.	(drinking adj behavi$3).mp.
23.	drinking behavior.mp.
24.	alcohol$.mp.
25.	alcoholism/
26.	(alcohol adj abuse).mp
27.	17 or 18 or 19 or 20 or 21 or 22 or 23 or 24 or 25 or 26
28.	*PNEUMONIA/
29.	bacterial pneumonia/ or infectious pneumonia/
30.	Mycoplasma pneumonia/
31.	COMMUNITY ACQUIRED PNEUMONIA/
32.	mycoplasma pneumon*.tw.
33.	(community-acquired pneumon* or community acquired pneumon*).tw.
34.	28 or 29 or 30 or 31 or 32 or 33
35.	16 and 27 and 34
**Web of Science search terms**	
(alcohol* OR alcoholic beverage OR alcohol consumption OR alcohol drinking OR alcohol use OR alcohol intake OR alcoholism OR alcohol abuse OR ethanol* OR ethanol concentration) AND (Pneumonia OR pneumon* OR bronchopneumon* OR bronchitis) AND (longitudinal * OR case control* OR Cohort* OR case-control OR observational)	

Table E2: Exploration of heterogeneity for alcohol consumption and CAP risk

Factor	Number of studies	Pooled RR (95% CI)	I^2	P value for subgroup differences
**Overall result**	14	1.83 [1.30, 2.57]	91%	-
**Study design**				0.07
Case control	9	2.16 [1.64, 2.85]	71%	
Cohort	4	1.56 [0.84, 2.91]	92%	
Cross sectional	1	1.20 [0.77, 1.85]	-	
**Methodological quality**				0.09
High quality (>6)	8	2.20 [1.40, 3.47]	93%	
Low quality (<6)	6	1.36 [0.99, 1.87]	57%	
**Alcohol consumption**				0.39
Alcohol vs no alcohol	6	1.61 [1.25, 2.08]	25%	
Alcohol vs lowest category of exposure	6	2.07 [1.24, 3.44]	95%	
**CAP ascertainment**				0.002
Clinical diagnosis	11	1.81[1.25, 2.61]	81%	
Death records	1	3.33 [2·92, 3.79]	0%	
**Geographic location**				0.0003
America	8	1.25 [1.00, 1.56]	56%	
Europe	5	3.03 [2.08, 4.43]	77%	
Australia	1	1.95 [1.08, 3.53]	-	
**Effect estimate**				0.03
Adjusted for confounders	10	2.05 [1.39, 3.01]	91%	
Unadjusted for confounders	4	1.20 [0.89, 1.62]	41%	
**Sex**				0.74
Men	3	2.10 [1.00, 4.41]	91%	
Women	3	1.71 [0.64, 4.57]	0%	

For peer review only

PRISMA Checklist

Section/topic	#	Checklist item	Reported on page #
**TITLE**			
Title	1	Identify the report as a systematic review, meta-analysis, or both.	1
**ABSTRACT**			
Structured summary	2	Provide a structured summary including, as applicable: background; objectives; data sources; study eligibility criteria, participants, and interventions; study appraisal and synthesis methods; results; limitations; conclusions and implications of key findings; systematic review registration number.	1
**INTRODUCTION**			
Rationale	3	Describe the rationale for the review in the context of what is already known.	1
Objectives	4	Provide an explicit statement of questions being addressed with reference to participants, interventions, comparisons, outcomes, and study design (PICOS).	1,2
**METHODS**			
Protocol and registration	5	Indicate if a review protocol exists, if and where it can be accessed (e.g., Web address), and, if available, provide registration information including registration number.	2
Eligibility criteria	6	Specify study characteristics (e.g., PICOS, length of follow-up) and report characteristics (e.g., years considered, language, publication status) used as criteria for eligibility, giving rationale.	2
Information sources	7	Describe all information sources (e.g., databases with dates of coverage, contact with study authors to identify additional studies) in the search and date last searched.	2
Search	8	Present full electronic search strategy for at least one database, including any limits used, such that it could be repeated.	2
Study selection	9	State the process for selecting studies (i.e., screening, eligibility, included in systematic review, and, if applicable, included in the meta-analysis).	2
Data collection process	10	Describe method of data extraction from reports (e.g., piloted forms, independently, in duplicate) and any processes for obtaining and confirming data from investigators.	3
Data items	11	List and define all variables for which data were sought (e.g., PICOS, funding sources) and any assumptions and simplifications made.	3

For peer review only

Risk of bias in individual studies	12	Describe methods used for assessing risk of bias of individual studies (including specification of whether this was done at the study or outcome level), and how this information is to be used in any data synthesis.	3
Summary measures	13	State the principal summary measures (e.g., risk ratio, difference in means).	3
Synthesis of results	14	Describe the methods of handling data and combining results of studies, if done, including measures of consistency (e.g., I^2) for each meta-analysis.	3,4

Section/topic	#	Checklist item	Reported on page #
Risk of bias across studies	15	Specify any assessment of risk of bias that may affect the cumulative evidence (e.g., publication bias, selective reporting within studies).	3
Additional analyses	16	Describe methods of additional analyses (e.g., sensitivity or subgroup analyses, meta-regression), if done, indicating which were pre-specified.	4
**RESULTS**			
Study selection	17	Give numbers of studies screened, assessed for eligibility, and included in the review, with reasons for exclusions at each stage, ideally with a flow diagram.	4
Study characteristics	18	For each study, present characteristics for which data were extracted (e.g., study size, PICOS, follow-up period) and provide the citations.	4,5,6
Risk of bias within studies	19	Present data on risk of bias of each study and, if available, any outcome level assessment (see item 12).	5
Results of individual studies	20	For all outcomes considered (benefits or harms), present, for each study: (a) simple summary data for each intervention group (b) effect estimates and confidence intervals, ideally with a forest plot.	7
Synthesis of results	21	Present results of each meta-analysis done, including confidence intervals and measures of consistency.	7
Risk of bias across studies	22	Present results of any assessment of risk of bias across studies (see Item 15).	7
Additional analysis	23	Give results of additional analyses, if done (e.g., sensitivity or subgroup analyses, meta-regression [see Item 16]).	7, 8
**DISCUSSION**			
Summary of evidence	24	Summarize the main findings including the strength of evidence for each main outcome; consider their relevance to key groups (e.g., healthcare providers, users, and policy makers).	8
Limitations	25	Discuss limitations at study and outcome level (e.g., risk of bias), and at review-level (e.g., incomplete retrieval of identified research, reporting bias).	8

For peer review only

Conclusions	26	Provide a general interpretation of the results in the context of other evidence, and implications for future research.	9
**FUNDING**			
Funding	27	Describe sources of funding for the systematic review and other support (e.g., supply of data); role of funders for the systematic review.	9

For peer review only

BMJ Open

Alcohol and the risk of pneumonia: A systematic review and meta-analysis

Journal:	*BMJ Open*
Manuscript ID	bmjopen-2018-022344.R1
Article Type:	Research
Date Submitted by the Author:	29-May-2018
Complete List of Authors:	Simou, Evangelia; university of Nottingham, Epidemiology and Public Health/Clinical Science Building<br>Britton, John; University of Nottingham/ UK Centre for Tobacco and Alcohol Studies, Division of Epidemiology<br>Leonardi-Bee, Jo; Uni Nottingham, Epidemiology and Public Health/ Clinical Science Building
**Primary Subject Heading**:	Respiratory medicine
Secondary Subject Heading:	Respiratory medicine, Epidemiology
Keywords:	Respiratory infections < THORACIC MEDICINE, Epidemiology < THORACIC MEDICINE, Infectious disease/HIV < NEUROLOGY

SCHOLARONE™
Manuscripts

Alcohol and the risk of pneumonia: A systematic review and meta-analysis

Evangelia Simou[1], John Britton[1], Jo Leonardi-Bee[1]

[1]UK Centre for Tobacco and Alcohol Studies, Division of Epidemiology and Public Health, University of Nottingham, Hucknall Road, Nottingham NG5 1PB

Address for Correspondence: Evangelia Simou, PhD student, Division of Epidemiology and Public Health, University of Nottingham, Hucknall Road, Nottingham NG5 1PB, Tel: +44 115 82 31388 Fax: +44 115 82 31337, Email: Evangelia.simou@nottingham.ac.uk

John Britton, Professor of Epidemiology; Director, UK Centre for Tobacco & Alcohol Studies, Faculty of Medicine & Health Sciences

Jo Leonardi- Bee, Professor of Medical Statistics & Epidemiology, Faculty of Medicine & Health Sciences

Word Count: 3683

For peer review only

Abstract

Objective: A systematic review and meta-analysis to estimate the magnitude of the association between alcohol consumption and the risk of community acquired pneumonia (CAP) in adults was undertaken.
Design: Systematic review and meta-analysis
Methods: Comprehensive searches of Medline, EMBASE and Web of Science were carried out to identify comparative studies of the association between alcohol intake and CAP between 1985 and 2017. Reference lists were also screened. A random effects meta-analysis was used to estimate pooled effect sizes. A dose response meta-analysis was also performed.
Results: We found 17 papers eligible for inclusion in the review, of which 14 provided results which could be pooled. Meta-analysis of these 14 studies identified a 83% increased risk of CAP among people who consumed alcohol, or in higher amounts, relative to those who consumed no, or lower amounts of alcohol respectively (RR= 1.83, 95% CI: 1.30-2.57). There was substantial between-study heterogeneity, which was attributable in part to differences in study continent, adjustment for confounders, and pneumonia diagnosis (clinical vs death). Dose-response analysis found that for every 10-20 grams higher alcohol intake per day, there was 8% increase in the risk of CAP.
Conclusions: The findings suggest that alcohol consumption increases the risk of CAP. Therefore, strengthening policies to reduce alcohol intake would be likely to reduce the incidence of CAP.

Key words: alcohol consumption; pneumonia; systematic review; meta-analysis; dose response analysis.

Strengths and limitations of this study

- This study represents a comprehensive review of the global literature with no language restrictions, whilst adhering to PRISMA and MOOSE guidelines.
- Heterogeneity was explored using subgroup analysis based on *a priori* defined factors.
- A dose response analysis of alcohol consumption was also performed.
- Confounding as a result of the existence of other factors that were not usually adjusted for in the included studies (e.g. socioeconomic status, malnutrition) could not be explored

INTRODUCTION

Pneumonia is a major cause of global morbidity and mortality. In 2014 in the United States, pneumonia (including influenza) was the eighth leading cause of death (1) and according to World Health Organization, in 2015 pneumonia was responsible for 16% of all deaths in children aged under 5 years (2). Community acquired infections are the most common cause of pneumonia, and with an annual incidence in Europe and North America of between 5 to 11 cases per thousand adults (3), community acquired pneumonias (CAP) account for an annual total of 4 million deaths annually (4). Globally, *Streptococcus pneumoniae* is the most common pathogen causing CAP (5). The annual incidence of community-acquired pneumonia requiring hospitalization among US adults is 24.8 cases per 10,000 adults; with highest incidence especially in oldest people (6). Patients with severe CAP admitted to European intensive care units have a mortality rate of 27% at six months (7).

For peer review only - http://bmjopen.bmj.com/site/about/guidelines.xhtml

Pneumonia is more common with increasing age (8, 9), among people who smoke (10-12) have a low body mass index (13) or have comorbidities including: other respiratory disease (12, 14), cardiovascular disease (14), stroke (14), dementia (11, 14) liver, or renal disease (14). Alcohol consumption is a potential risk factor for pneumonia. There are several possible mechanisms to explain the observation that alcohol consumption increases the risk of pneumonia, including the sedative properties of alcohol which can reduce oropharyngeal tone, leading to an increased risk of aspiration of microbes. Furthermore, high levels of alcohol intake can modify alveolar macrophage function, hence diminishing pulmonary defence against infection (15, 16). Also, high alcohol consumption is often associated with malnutrition (17) as it interacts with nutrient metabolism and utilization (18), resulting in the impairment of immunity and increases CAP risk.

To date however, evidence on the association between alcohol consumption and CAP is limited. A systematic review and meta-analysis published in 2010, using evidence published before August 2009, found a 6% increase in the risk of pneumonia per standard drink of 12 g of pure alcohol per day, but the number of studies reviewed (five) was small (19). However, there is an increase in the interest on this topic and also several studies have been published in the past nine years. For this reason we have carried out a systematic review and meta-analysis, to quantify the association between alcohol consumption and risk of CAP.

METHODS

The systematic review and meta-analysis was carried out in adherence with PRISMA (20) and MOOSE (21) guidelines. The protocol was published in the National Institute for Health Research International prospective register of systematic reviews (PROSPERO) under the registration number: 42015029910.

Patient and Public Involvement

No patients or public were involved in this review

Inclusion criteria

The PICO criteria were used for the eligibility of the articles based on type of study design, type of population, type of exposure and outcome. We included all comparative study designs (longitudinal, cohort, case-control, and cross sectional) assessing the association between alcohol intake and the risk of CAP in generally representative adult populations (>=18 years), and therefore excluded studies of selected populations such as people with HIV, Hepatitis B, or C virus infection; and those with hospital-acquired pneumonia. Where possible, we also analysed the association between alcohol consumption and the occurrence of pneumonia due to specific organisms (for example, *Streptococcus pneumonia*).

Exposure ascertainment

Alcohol consumption defined either by self-report (interview or questionnaire) or using medical records. Also, alcohol use corresponded to drinking levels (low, moderate, heavy, and alcoholism) or to frequency measures (grams/units/drinks per day/week).

Outcome ascertainment

Community acquired pneumonia diagnosis based: on a clinical diagnosis (chest x-ray, blood test), physician diagnosis and medical records including ICD codes or self-report.

Search strategy

For peer review only - http://bmjopen.bmj.com/site/about/guidelines.xhtml

Comprehensive search strategies were applied to the Medline (via Ovid), EMBASE (via Ovid), and Web of Science databases for the period from December 1985 to December 2017. We used search filters for observational study designs (22) and search terms for both outcome and exposure developed from relevant Cochrane Review groups (23). When searching, Medical Subject Headings (MeSH) terms were used for Medine and Embase; whereas free text words were used for Web of Science. The Medline search filters were the following: [exp Alcohol-Related Disorders/ OR Alcohol Drinking/ OR (alcohol adj3 (drink$ ORor intoxicat$ OR use$ OR abus$ OR misus$ OR-risk$ OR consum$ OR withdraw$ OR detox$ OR treat$ OR therap$ OR excess$ OR reduc$ OR cessation OR intervention$)).tw. OR (drink$ adj3 (excess OR heavy OR heavily OR harm OR harmful OR hazard$ OR binge OR problem$)).tw. OR alcoholic$.tw.] AND [exp Respiratory Tract Infections/ OR (acute respiratory infection*.tw.) OR (lower respiratory infection*.tw.) OR (lower respiratory tract infection*.tw.) OR exp Pneumonia/ OR (pneumon* OR bronchopneumon* OR pleuropneumon*).tw. OR exp Bronchitis/ OR (bronchit* OR bronchiolit*).tw].The full search strategy is presented (see Table E1 in the online data supplement). Reference lists of included studies were also screened in order to identify further potentially eligible studies. No language limitation was imposed and where necessary papers were translated into English. Where there was more than one report of findings from the same population (for example an abstract and then a full paper), the most recently published version of the study was used. Screening of titles and abstracts, as well as the full text, was conducted independently by two reviewers (ES and JL-B). Any disagreements were resolved thought discussion, or with the help of the third reviewer (JB).

Data extraction

Two reviewers (ES and JL-B) independently extracted data using a previously piloted form (see Table E2 in the online data supplement), which included the following information: author, year, study design, definitions of exposure (alcohol) and outcome (community acquired pneumonia), geographic location, reference population, and adjustment for confounders.

For categorical measures of alcohol drinking, where possible we compared any alcohol consumption with no alcohol consumption (reference group), or else used the lowest exposed category as the reference group. Also, in the main analysis, categorical measures of alcohol consumption were further defined as levels of consumption: light, moderate, heavy, binge and alcoholism. Grams of daily alcohol consumption were used as a standard measure, defining: one drink as 0.6 ounces, 14.0 grams, or 1.2 tablespoons of pure alcohol (24). Where possible, we followed the CDC guidelines for the definition of heavy drinking as a weekly consumption of 15, or more drinks for men, and 8 or more drinks for women; binge drinking as 5, or more drinks during a single occasion for men, or 4 or more for women; and excessive drinking as the presence of either binge or heavy drinking (24). The Dietary Guidelines for Americans defines moderate alcohol drinking as the daily consumption of up to one drink for women and two drinks for men (25). Otherwise we accepted the definitions of alcohol that the included studies used.

Quality assessment

Two authors (ES and JL-B) independently assessed the methodological quality of the included studies using the Newcastle-Ottawa Quality Scale (26). In the process of the quality assessment of each article a maximum score of nine stars can be obtained; whereas studies

For peer review only - http://bmjopen.bmj.com/site/about/guidelines.xhtml

with lower quality obtain fewer stars. In case of a cohort study the cohort study criteria were used; whereas for case control studies the case control criteria were used. However for a cross sectional study a modified version of the case control study criteria was used and in this case a maximum of 7 stars was given. All studies, irrespective of their design, were considered to be of high quality if they obtained a score of ≥6 stars. Discrepancies were resolved through discussion and consensus. We did not attempt to assess the methodological quality for studies published only in abstract form.

Statistical analysis

Relative measures of risk were extracted as odds ratios (OR), relative risks (RR) or hazard ratios (HR) with 95% confidence intervals. Where available, we used measures of risk adjusted for smoking and socioeconomic status and extracted results separately for men and women. Where raw data were extracted from studies, we estimated ORs for case control studies and RRs for longitudinal, cohort and cross sectional studies. Where exposure to alcohol was reported using quantiles, or categories, we extracted adjusted effect measures relating to a comparison of the highest to the lowest exposure group.

The pooled relative risk and the 95% CI were estimated through pooling ORs and RRs together, since it was assumed that these two measures of effect would be similar due to the outcome measure being uncommon (prevalence < ~10%)(27). However, HRs were not pooled with other effect measures. Meta-analysis was conducted, based on the DerSimonian and Laird's random effects model, to pool the results from the individual studies.

Heterogeneity between studies was quantified using I^2 statistics (28); and explored using subgroup analyses according to study quality, study design, adjustment for confounders, alcohol reference group (no alcohol vs lowest exposed category), CAP diagnosis (clinical diagnosis vs death records), geographical location (Low and Middle Income Countries versus High Income Countries) and measure of effect estimated (ORs vs RRs). Funnel plots were used as a visual aid to detect publication bias and where data for at least ten studies were available we formally assessed publication bias using Egger's asymmetry test. We performed all analyses using Stata (Version 14) and Review Manager (Version 5.3). All p-values <0.05 were deemed to represent statistical significance.

Dose response assessment

To assess the evidence for causality, we applied a modified version of Hill's criteria to assess causation (29) on strength of association, consistency, temporality, biological gradient and plausibility. To assess the biological gradient criterion we performed a random effects dose-response meta-analysis (30, 31), where we assumed a linear dose- response relation and allowed for study level correlations across the categories of quantities of alcohol. The dose-response relation between alcohol consumption and CAP was analysed using the subgroup of studies including at least three different categories of exposure, standardized for analysis to grams per day, and where appropriate using the midpoint of categories defined by ranges of intake. If the highest exposure category was open-ended, we took the highest category midpoint to be the lower bound plus 1.2 times the lower boundary (32). When available we included results for men and women separately.

Separate dose-response meta- analyses were conducted for cohort/ longitudinal and case control/ cross sectional studies. Dose categories relating to quantities of alcohol were created to equate to 10-20 grams of pure alcohol per day (approximately one drink per day);

For peer review only - http://bmjopen.bmj.com/site/about/guidelines.xhtml

where studies reported categories which contained the same dose ranges we collapsed these into a single dose category through estimating a pooled effect estimates based on a fixed effect meta- analysis model. Where necessary, effect estimates and 95% CI were back calculated from floated to conventional confidence intervals to enable comparisons to be made to the reference group (non- drinkers or the lowest exposed category) (33).

RESULTS

The searches identified a total of 4589 studies published between December 1985 and December 2017, of which 17 were eligible for inclusion in the systematic review (Figure 1). The characteristics of the 17 included studies are presented in Table 1. A total population of 287,184 people was included in our review. Seven studies used a cohort, or longitudinal design (10, 34-39), nine used a case control design (11, 40-47) and one used a cross sectional design(48). Eight studies were conducted in America (10, 11, 38, 39, 45-48); five in Europe (36, 40, 42-44), two in Asia (34, 35) and two in Australia (37, 41). Three studies reported separate estimates of the association between alcohol and CAP for men and women (10, 40, 43), and 12 studies reported effect estimates adjusted for confounders (10, 34, 35, 38, 40, 42-48).

The majority of studies assessed alcohol consumption by self-report, based either on a standardized questionnaire, or on an interview while five studies used reported intake data from medical records (11, 36, 39, 45, 46). The reference group for nine studies comprised people who never consumed alcohol (10, 34, 35, 37, 41, 43, 44, 46, 47); whereas the reference group for the remaining eight studies comprised people who consumed the lowest quantity of alcohol(11, 36, 38-40, 42, 45, 48).

Seven studies ascertained CAP using a clinical diagnosis; and five of these used chest x-ray radiography (41-44, 47). A further seven studies ascertained CAP using ICD codes (34, 35, 37, 39, 40, 45) and medical records (45) and two studies used self-report interview (38, 48). The remaining study ascertained CAP via physician diagnosis using medical records (10).

The methodological quality of the case control, cohort and cross sectional studies ranged from five to eight, with a median score of six. Ten studies were deemed to be of high quality (>6 score) (10, 34, 36-38, 40, 42, 44-46); whereas lower scores tended to arise from failure to adjust for confounders, or using self-reported methods to ascertain alcohol consumption. The results of the quality assessment are presented in detail in Table 2.

For peer review only

For peer review only - http://bmjopen.bmj.com/site/about/guidelines.xhtml

Study & Year	Study design	Geographical location	Alcohol ascertainment	Alcohol definition	CAP ascertainment	Confounders adjusted	Effect estimate
Almirall 1999 (44)	Case control	Europe/Spain	Self-report/questionnaire	Quartiles of alcohol intake >35·3 versus 0 (grams/day)	Clinically suspected and chest radiography	Age, sex, municipality	Odds ratio
Almirall 2008 (43)	Case control	Europe/Spain	Self-report /questionnaire	Quartiles of alcohol intake(grams/day) Men: >80 versus 0 Women: >40 versus 0	Clinically suspected and chest radiography	Age, sex, primary care practice	Odds ratio
Baik 2001 (10)	Cohort	America/US	Self-report/questionnaire	Men: >30 versus never Women: >30 versus never (Grams/day)	Physician diagnosis/Medical records	Age, smoking status, BMI, quintile of metabolic equivalent	Relative risk
Breitling 2016 (38)	Cohort	America/US	Self-report/questionnaire	Quartiles of alcohol intake Men: >20 versus ≤ 20 Women: >10 versus 0 (grams/day)	Self-report questionnaire	Age, sex, smoking, BMI, diabetes mellitus, stroke, congestive heart failure, cancer	Relative risk
Clough 2003 (41)	Case control	Australia	Self-report/interview	Alcohol yes versus alcohol no	Clinically suspected/ X-ray findings	-*	Odds ratio
Fernandez-Sola 1995 (42)	Case control	Europe/Spain	Self-report/ Interview & questionnaire	High intake (men: >100g, women:>80g) versus lower intake (grams/day 2 years before submission)	Clinically suspected/ Chest X-ray	Liver cirrhosis, smoking, COPD, diabetes, heart failure, malnutrition	Odds ratio
Innoue 2007 (35)	Cohort	Asia/Japan	Self-report/questionnaire	Current versus never drinking	Mortality ICD codes	Age and history of diabetes mellitus	Hazard ratio
Jackson 2009 (45)	Case control	America/US	Medical records	Current alcoholism vs no alcoholism	ICD9 codes	Age, sex, pneumonia-free person-time	Odds ratio
Koivula 1994(36)	Cohort	Europe/Finland	Medical records	Alcoholism vs no alcoholism	Medical records	Age, sex, chronic conditions	Relative risk
Lipsky 1986 (11)	Case control	America/US	Medical records	Heavy versus moderate (drinks/day)	Clinically suspected	-*	Relative risk
Loeb 2009 (47)	Case control	America/US	Self-report /questionnaire	Alcohol yes (previous 12 months) versus alcohol no(grams/month)	Clinically suspected and chest radiography	Multivitamins, smoking, history of gas and fumes exposure	Odds ratio
Phung 2013(37)	Cohort	Australia	Self-report/questionnaire	Alcohol yes versus alcohol no	Hospital records -ICD codes	-*	Hazard

For peer review only - http://bmjopen.bmj.com/site/about/guidelines.xhtml

For peer review only

							ratio
Quraishi 2013(48)	Cross sectional	America/US	Self-report/interview	alcohol consumption (≤30 versus >30 drinks per month)	Self-report interview	-*	Relative risk
Shen 2013 (34)	Cohort	Asia/China	Self-report/interview	Excessive versus never drinkers (units/week)	Mortality ICD codes	Age, Sex, education, housing, monthly expenditure, smoking, BMI, exercise, health status	Hazard ratio
Watt 2007 (46)	Case control	America/US	Medical records	Alcoholism/ alcohol use versus no use of alcohol	Clinically suspected Pneumococcal isolation in patient from sterile body fluid	Smoking, BMI, electricity/ indoor plumbing in home, living with unvaccinated child, unemployed, wood/coal, smoke	Odds ratio
Yende 2013 (39)	Cohort	America/US	Medical records	Alcohol abuse vs no alcohol abuse	ICD-9 codes	-*	Relative risk
Zaridze 2009 (40)	Case control	Europe/Russia	Self-report interview	≥3bottles(per week) versus <=0·5 bottles of vodka	ICD codes Death records	Age, city, and smoking	Relative risk

Table 1. Characteristics of the included studies

* Crude analysis reported

For peer review only - http://bmjopen.bmj.com/site/about/guidelines.xhtml

Table 2. Quality assessment- Newcastle Ottawa scale

Study, Year	Stars number			
	Selection†	Comparability‡	Exposure§	Overall
Almirall 1999 (44)	4	1	1	6/9
Almirall 2008 (43)	3	1	1	5/9
Baik 2001 (10)	4	2	2	8/9
Breitling 2016 (38)	3	2	2	5/9
Clough 2003 (41)	4	0	1	5/9
Fernandez-Sola 1995 (42)	3	2	1	6/9
Innoue 2007 (35)	3	1	1	5/9
Jackson 2009 (45)	4	1	1	6/9
Koivula 1994(36)	4	1	3	8/9
Lipsky 1986 (11)	3	0	2	5/9
Loeb 2009 (47)	2	2	1	5/9
Phung 2013(37)	3	0	3	6/9
Quraishi 2013(48)	1	0	1	2/6
Shen 2013 (34)	3	2	3	8/9
Watt 2007 (46)	4	2	1	7/9
Yende 2013 (39)	4	0	2	6/9
Zaridze 2009 (40)	3	2	1	6/9

Meta-analysis findings

Fourteen of the 17 included studies provided data from which pooled relative risks could be estimated, and a pooled analysis of these studies found the risk of CAP to be significantly increased in people who consumed alcohol at all, or in higher amounts, relative to those who consumed no, or lower amounts of alcohol respectively (pooled RR= 1.83, 95% CI 1.30 to 2.57, I^2= 91%, Figure 2). There was no evidence of publication bias detected visually via a funnel plot (see Figure E1 in the online data supplement), and statistically via Egger's asymmetry test (p= 0.596).

Subgroup analyses exploring the reason for heterogeneity in the meta-analysis of these 14 studies are presented in the Supplementary material (see Table E3). Heterogeneity was not explained by study design (case control, longitudinal/cohort, cross sectional; p for subgroup differences=0.07), methodological quality (high versus low; p=0.09) or gender (male versus female; p=0.74). However, significant differences were found according to adjustment for confounders (adjusted versus unadjusted; p=0.03), continent of study (America, Europe, Australia; p=0.0003), and ascertainment of CAP (clinical diagnosis vs death records; p=0.002). Furthermore no difference was found for studies presented OR estimates compared to studies presented RR estimates (p for subgroup differences=1.00).

Additionally, no significant differences were found by the definition of the reference group for alcohol consumption (p=0.39; Figure 2). However, high heterogeneity (I^2=95%) was detected within the second subgrouping which used the lowest category of exposure as the reference group, where the following definitions were used: no alcoholism (36, 45),no alcohol abuse (39), moderate drinking (11), ≤30 drinks/month(48), ≤ 0.5 bottles of vodka(40); <100gr/day for men and <80 gr/day for women (42), and <20 gr/day and <10 gr/day for men and women respectively (38); however, the gradient of exposure did not seem to be related to the magnitude of effect.

For peer review only - http://bmjopen.bmj.com/site/about/guidelines.xhtml

A sensitivity analysis restricted to the six studies which provided smoking-adjusted estimates found a larger magnitude of effect compared to the main analysis (pooled RR= 2.01, 95% CI 1.25 to 3.23, I^2=93%, 6 studies). Similarly the studies provided age-adjusted effect estimated found a risk of 1.90 (pooled RR= 1.90, 95% CI 1.20 to 3.02, I^2=93%, 7 studies).

The remaining three studies presented effect estimates as hazard ratios (34, 35, 37), and a pooled analysis of these studies estimated a hazard ratio for CAP in relation to alcohol consumption of 0.90 (pooled HR= 0.90, 95%CI: 0.79 to 1.03, I^2=0, 3 studies).

Two studies assessing the effect of alcohol on pneumococcal disease specific strains of pneumonia were identified (11, 46) . A pooled analysis of these studies found that there was more than a doubling of risk of *Streptococcus pneumoniae* CAP in people who consumed alcohol (RR= 2.16, 95% CI 1.05 to 4·48, I^2=42%)

Biological gradient meta-analysis

Five of the included studies provided data enabling a dose-response meta-analysis (10, 40, 41, 43, 44); of which: one used a cohort design (data reported separately for men and women) and four were case-control studies. A pooled analysis of the dose-response data from the cohort study found no significant gradient in the quantity of alcohol associated with the risk of CAP (p for trend=0.136). In contrast, the pooled analysis of the dose-response data from the four case control studies indicated that there was a significant gradient in the quantity of alcohol associated with a 8% increase in the risk of CAP for every 10-20 grams of pure alcohol consumed per day (equivalent to 1 drinks/day) (pooled RR= 1.08, 95% CI 1.06 to 1.09; p<0.0001; Figure 3).

DISCUSSION

Alcohol consumption is a recognised and avoidable risk factor for a range of diseases and injuries, including neuropsychiatric conditions, gastrointestinal and cardiovascular disease, cancer, suicide, violence and tuberculosis (49). To date however the association between alcohol consumption and pneumonia risk has attracted relatively little attention.

Summary of the findings

This meta-analysis of studies published over the past 30 years- demonstrates a clear and statistically significant relation between alcohol consumption and the risk of community acquired pneumonia. The effect was strong, with a 1.8 fold increase in risk among those who consumed alcohol at all, or in higher amounts, relative to those who consumed no, or lower amounts of alcohol respectively and significantly related to level of intake, with no evidence of publication bias. The dose response analysis indicated that consuming drinks that contain 10-20 grams of alcohol per day was linked to an 8% increased risk of acquiring community acquired pneumonia. Furthermore, the findings of the subgroup analysis indicated significant differences in the risk of pneumonia according to continent of the study; with Europe having the highest rate (threefold) for CAP risk.

Strengths and limitations

For peer review only - http://bmjopen.bmj.com/site/about/guidelines.xhtml

For peer review only

This study represents a comprehensive review of the global literature with no language restriction, making this analysis the most complete to date, and our findings likely to be generalizable. There was significant heterogeneity between the studies in our analysis, but our subgroup analyses indicate that this arose primarily from the continent in which the study was carried out (America, Europe, Australia); adjustment for confounders; and the ascertainment of CAP (death vs clinical diagnosis). Misclassification bias arising from inclusion of non-drinkers in the lowest category of alcohol intake in some studies can be another possible limitation in our review, but will result in a more conservative estimate of effect. A dose response relationship was identified. However the included studies did not report dose response relations separately for men and women, so we are unable to carry out a comparative analysis. Furthermore, confounding as a result of the existence of other factors that were not usually adjusted for in the included studies (e.g. socioeconomic status, malnutrition) could not be explored.

Comparison with other studies

Our findings extend those of an earlier review and meta-analysis, carried out in 2010 (19). Another review focussed on risk factors for invasive pneumococcal diseases, indicated an elevated risk for invasive pneumococcal disease due to alcohol consumption in six of the four studies included in the meta-analysis model (50). Likewise, another recent meta-analysis indicated an elevated risk for invasive pneumococcal disease due to alcohol consumption in six of the four studies included in the meta-analysis model (51). Similarly our separate meta-analysis focused on pneumococcal infections including two of these studies, due to our eligibility criteria, showed an elevated risk for pneumococcal acquisition.

A previous systematic review and meta-analysis found that people with a daily alcohol consumption of either 24, 60, and 120 grams have a 12 %, 33% and 76 % increased risk of CAP respectively (19). Our dose response analysis generated a slightly less strong effect, of an 8% increase in risk per 10-20 grams of (pure) alcohol consumed per day.

A general systematic review published by Almirall et al in 2017 (52) focused on risk factors of community acquired pneumonia, but provided only a narrative summary of findings and stating that no definite conclusion could be drawn. In contrast, our review found evidence of a doubling in the risk of CAP in people who consumed alcohol. Furthermore, our demonstration of a significant exposure-response association increases the likelihood, given the strength of the observed association and its consistency across a range of subgroups, that the observed association is causal. Further evidence of causality arises from studies demonstrating that alcohol consumption impairs alveolar macrophages and increases carriage of pneumonia pathogens (15, 16, 53).

Clinical implications

The findings from the present review highlight the need to address high alcohol consumption as a means to prevent community acquired pneumonia. Clinicians managing patients with pneumonia could for example counsel reducing alcohol intake as a means to prevent further episodes; and those addressing high alcohol consumption in more general terms could add an increased risk of pneumonia as a further reason to reduce intake.

For peer review only - http://bmjopen.bmj.com/site/about/guidelines.xhtml

Our findings also have implications for public health: in Europe for example, the estimated annual costs of CAP are approximately €10.1 billion (54), might be reduced substantially by more pro-active clinical and public health measures to reduce alcohol consumption.

Conclusion

Our findings thus provide clear evidence that alcohol increases the risk of pneumonia. Informing people who drink alcohol of this risk, especially those who consume high levels of alcohol, both in clinical contacts and through public health policy, may therefore help to prevent this disease.

For peer review only

Contributors

ES, JB and JL-B designed the study and wrote the protocol. ES wrote the search strategy and undertook the literature searches, and wrote the draft of the manuscript. ES and JLB undertook study screening, data extraction, and quality assessment. ES undertook all data analysis, supervised by JL-B. All authors contributed to the interpretation of the findings. JB and JLB provided critical revisions to the article, and all authors approved the final version of the article to be published. ES acts as guarantor of the manuscript.

Conflicts of interest

None declared

Acknowledgments

The authors thank Erica Brasil, Magdalena Opazo-Breton and Yue Huang from the University of Nottingham for their help in translations

Funding: This work was supported by the Medical Research Council [grant number MR/K023195/1]; the UK Centre for Tobacco and Alcohol Studies (http://www.ukctas.net); and the British Heart Foundation, Cancer Research UK, the Economic and Social Research Council, and the National Institute of Health Research, under the auspices of the UK Clinical Research Collaboration, is gratefully acknowledged.

Ethics approval: Ethics approval was not required for this work

Data sharing: No additional data are available.

References

1. Murphy SL, Kochanek KD, Xu J, Arias E. Mortality in the United States, 2014. NCHS data brief. 2015;229:1-8.
2. World Health Organization, Pneumonia Fact Sheet;World Health Organization Report 2016, WHO: Geneva, Switzerland. 2016.
3. Rozenbaum M, Pechlivanoglou P, Van Der Werf T, Lo-Ten-Foe J, Postma M, Hak E. The role of Streptococcus pneumoniae in community-acquired pneumonia among adults in Europe: a meta-analysis. Eur J Clin Microbiol Infect Dis. 2013;32(3):305-16.
4. Ruuskanen O, Lahti E, Jennings LC, Murdoch DR. Viral pneumonia. The Lancet. 2011;377(9773):1264-75.
5. Woodhead M. Community-acquired pneumonia in Europe: causative pathogens and resistance patterns. Eur Respir J. 2002;20(36 suppl):20s-7s.
6. Jain S, Self WH, Wunderink RG, Fakhran S, Balk R, Bramley AM, et al. Community-acquired pneumonia requiring hospitalization among US adults. N Engl J Med. 2015;373(5):415-27.

7. Walden AP, Clarke GM, McKechnie S, Hutton P, Gordon AC, Rello J, et al. Patients with community acquired pneumonia admitted to European intensive care units: an epidemiological survey of the GenOSept cohort. Critical care. 2014;18(2):R58.
8. European Commission. Health statistics, Atlas on mortality in the European Union, Luxembourg: Office for Official Publications of the European Communities. 2008.
9. Jain S, Williams DJ, Arnold SR, Ampofo K, Bramley AM, Reed C, et al. Community-acquired pneumonia requiring hospitalization among US children. N Engl J Med. 2015;372(9):835-45.
10. Baik I, Curhan GC, Rimm EB, Bendich A, Willett WC, Fawzi WW. A prospective study of age and lifestyle factors in relation to community-acquired pneumonia in US men and women. Arch Intern Med. 2000;160(20):3082-8.
11. Lipsky BA, Boyko EJ, Inui TS, Koepsell TD. Risk factors for acquiring pneumococcal infections. Arch Intern Med. 1986;146(11):2179-85.
12. Kohlhammer Y, Schwartz M, Raspe H, Schäfer T. Risk factors for community acquired pneumonia (CAP). A systematic review. Deutsche medizinische Wochenschrift (1946). 2005;130(8):381-6.
13. Simet SM, Sisson JH. Alcohol's effects on lung health and immunity. Alcohol research: current reviews. 2015;37(2):199.
14. Vinogradova Y, Hippisley-Cox J, Coupland C. Identification of new risk factors for pneumonia: population-based case-control study. Br J Gen Pract. 2009;59(567):e329-e38.
15. Mehta AJ, Guidot DM. Pathophysiology Review Series: Alcohol Abuse, the Alveolar Macrophage, and Pneumonia. The American journal of the medical sciences. 2012;343(3):244.
16. Kershaw CD, Guidot DM. Alcoholic lung disease. Alcohol Research & Health. 2008;31(1):66-76.
17. Moss M. Epidemiology of sepsis: race, sex, and chronic alcohol abuse. Clin Infect Dis. 2005;41(Supplement_7):S490-S7.
18. Marsano L. Alcohol and malnutrition. Alcohol Research and Health. 1993;17(4):284.
19. Samokhvalov A, Irving H, Rehm J. Alcohol consumption as a risk factor for pneumonia: a systematic review and meta-analysis. Epidemiology and infection. 2010;138(12):1789-95.
20. Moher D, Liberati A, Tetzlaff J, Altman DG. Preferred reporting items for systematic reviews and meta-analyses: the PRISMA statement. Ann Intern Med. 2009;151(4):264-9.
21. Stroup DF, Berlin JA, Morton SC, Olkin I, Williamson GD, Rennie D, et al. Meta-analysis of observational studies in epidemiology: a proposal for reporting. Journal of American Medical Association. 2000;283(15):2008-12.
22. SIGN. Search Filters, Obseravtional studies, Available from: http://www.sign.ac.uk/methodology/filters.html#obs Accessed 4 December 2015.
23. Cochrane Library. Available from: http://www.cochranelibrary.com/ Accessed 4 December 2015.
24. Centers for Disease Control and Prevention (CDC). Alcohol & Public Health, Fact Sheets - Alcohol Use and Your Health, http://www.cdc.gov/alcohol/fact-sheets/alcohol-use.htm Accessed 10 December 2015.
25. U.S Department of Health and Human Services and U.S Department of Agriculture. 2015 – 2020.Dietary Guidelines for Americans. 8th Edition, Washington, DC; 2015, https://health.gov/dietaryguidelines/2015/resources/2015-2020_Dietary_Guidelines.pdf. Accessed December 15, 2015.
26. Wells G, Shea B, O'connell D, Peterson J, Welch V, Losos M, et al. The Newcastle-Ottawa Scale (NOS) for assessing the quality of nonrandomised studies in meta-analyses http://www.ohri.ca/programs/clinical_epidemiology/oxford.asp Accessed 10 February 2016.
27. Deeks J. Swots corner: what is an odds ratio. Bandolier. 1996;3(3):6-7.
28. Higgins J, Thompson SG. Quantifying heterogeneity in a meta-analysis. Statistics in Medicine. 2002;21(11):1539-58.
29. Hill AB. The environment and disease: association or causation? : SAGE Publications; 1965.

For peer review only - http://bmjopen.bmj.com/site/about/guidelines.xhtml

30. Greenland S, Longnecker MP. Methods for trend estimation from summarized dose-response data, with applications to meta-analysis. Am J Epidemiol. 1992;135(11):1301-9.
31. Orsini N, Bellocco R, Greenland S. Generalized least squares for trend estimation of summarized dose-response data. Stata Journal. 2006;6(1):40.
32. Berlin JA, Longnecker MP, Greenland S. Meta-analysis of epidemiologic dose-response data. Epidemiology. 1993;4(3):218-28.
33. Orsini N. From floated to conventional confidence intervals for the relative risks based on published dose–response data. Computer methods and programs in biomedicine. 2010;98(1):90-3.
34. Shen C, Ni MY, Schooling CM, Chan WM, Lee SY, Lam TH. Alcohol use and death from respiratory disease in a prospective Chinese elderly cohort study in Hong Kong. Prev Med. 2013;57(6):819-23.
35. Inoue Y, Koizumi A, Wada Y, Iso H, Watanabe Y, Date C, et al. Risk and protective factors related to mortality from pneumonia among middleaged and elderly community residents: the JACC Study. J Epidemiol. 2007;17(6):194-202.
36. Koivula I, Sten M, Makela PH. Risk factors for pneumonia in the elderly. The American journal of medicine. 1994;96(4):313-20.
37. Phung DT, Wang Z. Risk of pneumonia in relation to body mass index in Australian Aboriginal people. Epidemiol Infect. 2013;141(12):2497-502.
38. Breitling LP, Saum K-U, Schöttker B, Holleczek B, Herth FJ, Brenner H. Pneumonia in the Noninstitutionalized Older Population: A prospective observational study over 11 years. Deutsches Ärzteblatt International. 2016;113(37):607.
39. Yende S, Alvarez K, Loehr L, Folsom AR, Newman AB, Weissfeld LA, et al. Epidemiology and long-term clinical and biologic risk factors for pneumonia in community-dwelling older Americans: analysis of three cohorts. Chest. 2013;144(3):1008-17.
40. Zaridze D, Brennan P, Boreham J, Boroda A, Karpov R, Lazarev A, et al. Alcohol and cause-specific mortality in Russia: a retrospective case-control study of 48 557 adult deaths. Lancet. 2009;373(9682):2201-14.
41. Clough AR, Wang Z, Bailie RS, Burns CB, Currie BJ. Case-control study of the association between kava use and pneumonia in eastern Arnhem and Aboriginal communities (Northern Territory, Australia). Epidemiol Infect. 2003;131(1):627-35.
42. Fernandezsola J, Junque A, Estruch R, Monforte R, Torres A, Urbanomarquez A. HIGH ALCOHOL INTAKE AS A RISK AND PROGNOSTIC FACTOR FOR COMMUNITY-ACQUIRED PNEUMONIA. Arch Intern Med. 1995;155(15):1649-54.
43. Almirall J, Bolibar I, Serra-Prat M, Roig J, Hospital I, Carandell E, et al. New evidence of risk factors for community-acquired pneumonia: a population-based study. Eur Respir J. 2008;31(6):1274-84.
44. J. Almirall IB, X. BalanzoÂ, C.A. GonzaÂ lez. Risk factors for community-acquired pneumonia in adults:

a population-based case±control study. Eur Respir J. 1999;13:349-55.
45. Jackson ML, Nelson JC, Jackson LA. Risk Factors for Community-Acquired Pneumonia in Immunocompetent Seniors. J Am Geriatr Soc. 2009;57(5):882-8.
46. Watt JP, O'Brien KL, Benin AL, McCoy SI, Donaldson CM, Reid R, et al. Risk factors for invasive pneumococcal disease among Navajo adults. Am J Epidemiol. 2007;166(9):1080-7.
47. Loeb M, Neupane B, Walter SD, Hanning R, Carusone SC, Lewis D, et al. Environmental Risk Factors for Community-Acquired Pneumonia Hospitalization in Older Adults. J Am Geriatr Soc. 2009;57(6):1036-40.
48. Quraishi SA, Bittner EA, Christopher KB, Camargo Jr CA. Vitamin D status and community-acquired pneumonia: results from the third National Health and Nutrition Examination Survey. PLoS ONE. 2013;8(11):e81120.
49. World Health Organization. Global status report on alcohol and health 2014.

For peer review only

For peer review only - http://bmjopen.bmj.com/site/about/guidelines.xhtml

50. Boé DM VR, Burnham EL, Moss M. . Alcohol abuse and pulmonary disease. . Journal of leukocyte biology. 2009 Nov 1;86(5):1097-104.
51. Cruickshank HC, Jefferies JM, Clarke SC. Lifestyle risk factors for invasive pneumococcal disease: a systematic review. BMJ Open. 2014;4(6):e005224.
52. Almirall J, Serra-Prat M, Bolíbar I, Balasso V. Risk Factors for Community-Acquired Pneumonia in Adults: A Systematic Review of Observational Studies. Respiration. 2017.
53. Dao TT, Liebenthal D, Tran TK, Ngoc Thi Vu B, Ngoc Thi Nguyen D, Thi Tran HK, et al. Klebsiella pneumoniae oropharyngeal carriage in rural and urban Vietnam and the effect of alcohol consumption. PLoS ONE. 2014;9(3):e91999.
54. Welte T, Torres A, Nathwani D. Clinical and economic burden of community-acquired pneumonia among adults in Europe. Thorax. 2012;67(1):71-9.

For peer review only

Table of contents

Table 1. Characteristics of the included studies.. 7
Table 2. Quality assessment- Newcastle Ottawa scale 8

Figure legend

Figure 1. Study selection……………………………………………………………………………………………………
Figure 2. Forest plot of alcohol consumption and risk of CAP; subgroup analysis based on reference group(never drinking versus lowest drinking category) ……………………………………..
Figure 3. Linear dose response meta-analysis for the association between alcohol intake categories (grams/day) and the risk of CAP …………………………………………………………………….

For peer review only - http://bmjopen.bmj.com/site/about/guidelines.xhtml

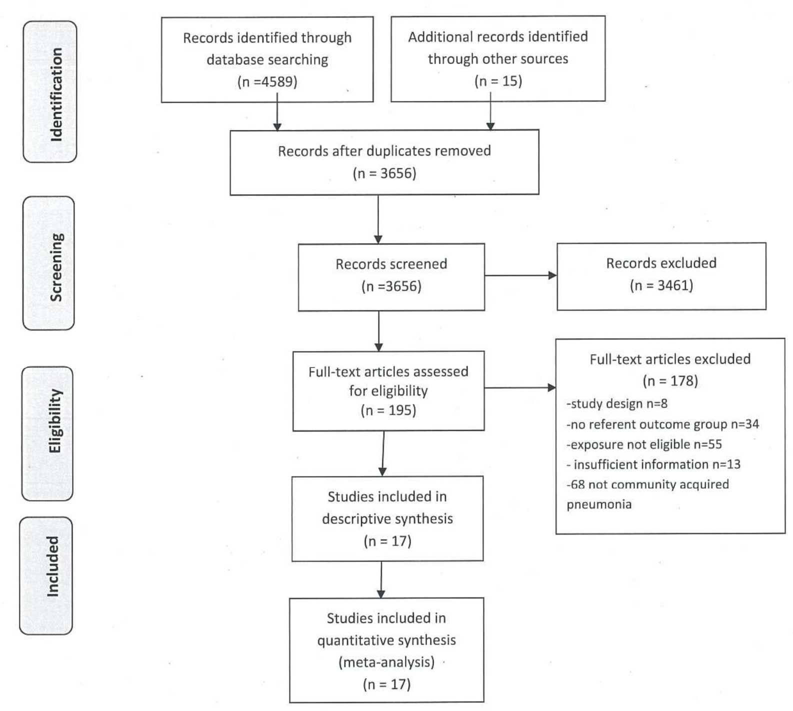

Figure 1. Study selection

209x298mm (300 x 300 DPI)

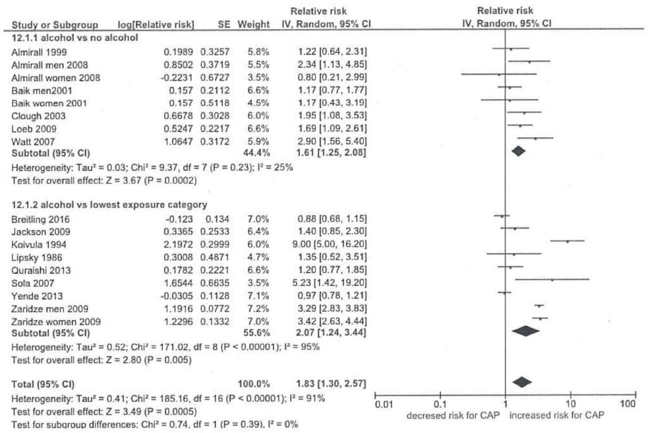

Figure 2. Forest plot of alcohol consumption and risk of CAP; subgroup analysis based on reference group(never drinking versus lowest drinking category)

209x298mm (300 x 300 DPI)

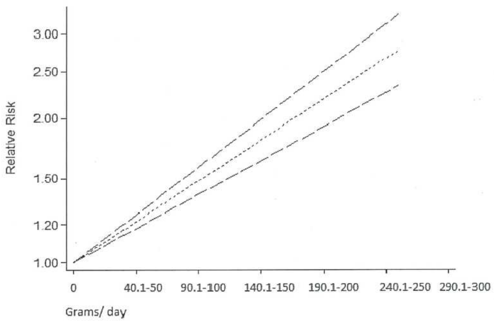

Figure 3. Linear dose response meta-analysis for the association between alcohol intake categories (grams/day) and the risk of CAP

209x298mm (300 x 300 DPI)

Alcohol and the risk of pneumonia: A systematic review and meta-analysis

Evangelia Simou, John Britton, Jo Leonardi-Bee

Online Data Supplement

**Table E1: Medline (via Ovid) and EMBASE (via Ovid) search terms for primary studies**
**Medline via Ovid search terms**
1. Epidemiologic studies/
2. Exp case control studies/
3. Exp cohort studies/
4. Case control.tw.
5. (cohort adj (study or studies)).tw.
6. Cohort analy$.tw.
7. (Follow up adj (study or studies)).tw.
8. (observational adj (study or studies)).tw.
9. Longitudinal.tw.
10. Retrospective.tw.
11. Cross sectional.tw.
12. Cross-sectional studies/
13. Or/1-12
14. exp Alcohol-Related Disorders/
15. Alcohol Drinking/
16. (alcohol adj3 (drink$ or intoxicat$ or use$ or abus$ or misus$ or risk$ or consum$ or withdraw$ or detox$ or treat$ or therap$ or excess$ or reduc$ or cessation or intervention$)).tw.
17. (drink$ adj3 (excess or heavy or heavily or harm or harmful or hazard$ or binge or problem$)).tw.
18. alcoholic$.tw.
19. 14 or 15 or 16 or 17 or 18
20. exp Respiratory Tract Infections/
21. acute respiratory infection*.tw.
22. lower respiratory infection*.tw.
23. lower respiratory tract infection*.tw.
24. exp Pneumonia/
25. (pneumon* or bronchopneumon* or pleuropneumon*).tw.
26. exp Bronchitis/
27. (bronchit* or bronchiolit*).tw.
28. 20 or 21 or 22 or 23 or 24 or 25 or 26 or 27
29. 13 and 19 and 28
**Embase via Ovid search terms**
1. Clinical study/
2. Case control study
3. Family study/
4. Longitudinal study/
5. Retrospective study/
6. Prospective study/
7. Randomized controlled trials/
8. 6 not 7
9. Cohort analysis/
10. (Cohort adj (study or studies)).mp.
11. (Case control adj (study or studies)).tw.
12. (follow up adj (study or studies)).tw.
13. (observational adj (study or studies)).tw.
14. (epidemiologic$ adj (study or studies)).tw.
15. (cross sectional adj (study or studies)).tw.
16. Or/1-5,8-15
17. substance-related disorders/
18. ((drug or substance) adj (Addict$ or abus$ or dependen$)).mp
19. (intoxicat$ or abstinen$ or withdrawal$).mp.
20. (excessive$ adj use$).mp.
21. (use$ adj disorder$).mp.

For peer review only

22.	(drinking adj behavi$3).mp.
23.	drinking behavior.mp.
24.	alcohol$.mp.
25.	alcoholism/
26.	(alcohol adj abuse).mp
27.	17 or 18 or 19 or 20 or 21 or 22 or 23 or 24 or 25 or 26
28.	*PNEUMONIA/
29.	bacterial pneumonia/ or infectious pneumonia/
30.	Mycoplasma pneumonia/
31.	COMMUNITY ACQUIRED PNEUMONIA/
32.	mycoplasma pneumon*.tw.
33.	(community-acquired pneumon* or community acquired pneumon*).tw.
34.	28 or 29 or 30 or 31 or 32 or 33
35.	16 and 27 and 34
**Web of Science search terms**	
(alcohol* OR alcoholic beverage OR alcohol consumption OR alcohol drinking OR alcohol use OR alcohol intake OR alcoholism OR alcohol abuse OR ethanol* OR ethanol concentration) AND (Pneumonia OR pneumon* OR bronchopneumon* OR bronchitis) AND (longitudinal * OR case control* OR Cohort* OR case-control OR observational)	

Table E2: Data extraction form

Reviewer name: **Study Author and Year:**

DESCRIPTION OF STUDY

Study Design	Cohort ☐ Prospective ☐ Retrospective ☐ Nested Case control ☐
Name of Cohort<br><br>Data collection years	Start date (year): End date (year):<br><br>Years of follow-up data:
Definition of Alcohol<br><br>(Any record of the number of drinks per day or gr of ethanol per day, number of drinks consumed annually, record of drinking levels: light, moderate and heavy drinking age since started alcohol consumption, specific alcohol drinks, alcoholism)	
Definition of health condition	Method of diagnosis:<br>Exposure Outcome Both
Setting (e.g. developed/non-developed, public/private health care, urban/rural)	
Country- European?	
Selection of controls	

For peer review only

(for nested case control studies only)	

PARTICIPANTS

Inclusion criteria	
Exclusion criteria	
Number entering into study (may not be recorded)	
Final number of participants evaluated	

For peer review only

DEMOGRAPHICS OF STUDY POPULATION

Age (mean, SD, range)	
Gender (n, % male)	
Other demographics reported (e.g. ethnicity, place of residence, occupation, education, socioeconomic)	

RESULTS

Outcome	Adjusted measure of effect with 95% CI (in preference)<br>Crude measure of effect with 95% CI<br>Raw numbers<br>P value
1.first outcome	Exposure:<br>Comparator:<br>Result:
2. second outcome	Exposure:<br>Comparator:<br>Result:

SOURCE OF FUNDING

(e.g. Government (NHS), voluntary/charity, pharmaceutical company)

LIMITATIONS

Identified by author	
Identified by review team	
Evidence gaps and/or recommendations for future research	

Table E3: Exploration of heterogeneity for alcohol consumption and CAP risk

Factor	Number of studies	Pooled RR (95% CI)	I^2	P value for subgroup differences
**Overall result**	14	1.83 [1.30, 2.57]	91%	-
**Study design**				0.07
Case control	9	2.16 [1.64, 2.85]	71%	
Cohort	4	1.56 [0.84, 2.91]	92%	
Cross sectional	1	1.20 [0.77, 1.85]	-	
**Methodological quality**				0.09
High quality (>6)	8	2.20 [1.40, 3.47]	93%	
Low quality (<6)	6	1.36 [0.99, 1.87]	57%	
**Alcohol consumption**				0.39
Alcohol vs no alcohol	6	1.61 [1.25, 2.08]	25%	
Alcohol vs lowest category of exposure	6	2.07 [1.24, 3.44]	95%	
**CAP ascertainment**				0.002
Clinical diagnosis	11	1.81[1.25, 2.61]	81%	
Death records	1	3.33 [2·92, 3.79]	0%	
**Geographic location**				0.0003
America	8	1.25 [1.00, 1.56]	56%	
Europe	5	3.03 [2.08, 4.43]	77%	
Australia	1	1.95 [1.08, 3.53]	-	
**Effect estimate**				0.03
Adjusted for confounders	10	2.05 [1.39, 3.01]	91%	
Unadjusted for confounders	4	1.20 [0.89, 1.62]	41%	
**Measure of effect estimate**				1.00
ORs	7	1.81 [1.38, 2.36]	25%	
RRs	7	1.81 [1.10, 2.99]	95%	
**Sex**				0.74
Men	3	2.10 [1.00, 4.41]	91%	
Women	3	1.71 [0.64, 4.57]	0%	

For peer review only

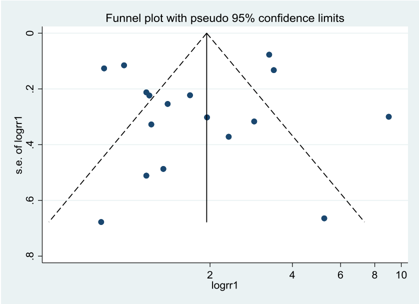

Figure E1: Funnel plot of alcohol vs no alcohol or lowest exposure to alcohol for studies presented the results as RRs.

PROSPERO International prospective register of systematic reviews

Review title and timescale

1 Review title

Give the working title of the review. This must be in English. Ideally it should state succinctly the interventions or exposures being reviewed and the associated health or social problem being addressed in the review.

Systematic review and meta-analysis of the effect of alcohol consumption on specific types of cancer and severe lung diseases

2 Original language title

For reviews in languages other than English, this field should be used to enter the title in the language of the review. This will be displayed together with the English language title.

3 Anticipated or actual start date

Give the date when the systematic review commenced, or is expected to commence.

01/12/2015

4 Anticipated completion date

Give the date by which the review is expected to be completed.

30/09/2018

5 Stage of review at time of this submission

Indicate the stage of progress of the review by ticking the relevant boxes. Reviews that have progressed beyond the point of completing data extraction at the time of initial registration are not eligible for inclusion in PROSPERO. This field should be updated when any amendments are made to a published record.

The review has not yet started √

Review stage	Started	Completed
Preliminary searches	Yes	No

For peer review only - http://bmjopen.bmj.com/site/about/guidelines.xhtml

Piloting of the study selection process	No	No
Formal screening of search results against eligibility criteria	No	No
Data extraction	No	No
Risk of bias (quality) assessment	No	No
Data analysis	No	No

Provide any other relevant information about the stage of the review here.

Review team details

6 Named contact

The named contact acts as the guarantor for the accuracy of the information presented in the register record.

Evangelia Simou

7 Named contact email

Enter the electronic mail address of the named contact.

msxes6@nottingham.ac.uk

8 Named contact address

Enter the full postal address for the named contact.

UK Centre for Tobacco and Alcohol Studies, Division of Epidemiology and Public Health, University of Nottingham, Clinical Sciences Building, Hucknall Road, Nottingham, NG5 1PB, UK

9 Named contact phone number

Enter the telephone number for the named contact, including international dialing code.

+44 (0) 115 82 31388

10 Organisational affiliation of the review

Full title of the organisational affiliations for this review, and website address if available. This field may be completed as 'None' if the review is not affiliated to any organisation.

The University of Nottingham

Website address:

http://nottingham.ac.uk/medicine/about/eph/index.aspx

11 Review team members and their organisational affiliations

Give the title, first name and last name of all members of the team working directly on the review. Give the organisational affiliations of each member of the review team.

Title	First name	Last name	Affiliation
Professor	John	Britton	Director, UK Centre for Tobacco & Alcohol Studies, Faculty of Medicine & Health Sciences,School of Medicine
Professor	Jo	Leonardi-Bee	UK Centre for Tobacco and Alcohol Studies, Faculty of Medicine & Health Sciences,School of Medicine,Univesrity of Nottingham

12 Funding sources/sponsors

Give details of the individuals, organizations, groups or other legal entities who take responsibility for initiating, managing, sponsoring and/or financing the review. Any unique identification numbers assigned to the review by the individuals or bodies listed should be included.

UK Centre for Tobacco & Alcohol Studies,(UKCTAS).

13 Conflicts of interest

List any conditions that could lead to actual or perceived undue influence on judgements concerning the main topic investigated in the review.

Are there any actual or potential conflicts of interest?

None known

14 Collaborators

Give the name, affiliation and role of any individuals or organisations who are working on the review but who are not listed as review team members.

Title	First name	Last name	Organisation details
Professor	Ian	Gilmore	

Review methods

15 Review question(s)

State the question(s) to be addressed / review objectives. Please complete a separate box for each question.

How does alcohol consumption impact on specific cancers and severe lung diseases on adults?

16 Searches

Give details of the sources to be searched, and any restrictions (e.g. language or publication period). The full search strategy is not required, but may be supplied as a link or attachment.

We will search the following electronic bibliographic databases: MEDLINE (via Ovid), EMBASE (via Ovid) and Web of Science. Studies published between 1985 and the date the searches are run will be sought. Emphasis will be given on the most recent studies. A ‘search diary’ will be kept giving details for the search strategy, including the names of the databases searched, the search terms used and the search results The search filter used by SIGN will be adopted to retrieve systematic reviews. Search terms for each health outcome will be developed from search strategies from relevant Cochrane Review groups. There will be no language restrictions.

17 URL to search strategy

If you have one, give the link to your search strategy here. Alternatively you can e-mail this to PROSPERO and we will store and link to it.

I give permission for this file to be made publicly available
Yes

18 Condition or domain being studied

Give a short description of the disease, condition or healthcare domain being studied. This could include health and wellbeing outcomes.
A full list of the outcomes being assessed are given under 'Primary outcomes' below.

19 Participants/population

Give summary criteria for the participants or populations being studied by the review. The preferred format includes details of both inclusion and exclusion criteria.
Inclusion: Adults aged 18 and over. Exclusion: Adults 18 years and older who do not consume alcohol.

20 Intervention(s), exposure(s)

Give full and clear descriptions of the nature of the interventions or the exposures to be reviewed
All studies which have assessed the effect of alcohol consumption defined as ever alcohol drinkers, ex-or former drinkers will be included. For the alcohol to include all drinking levels: light, moderate and heavy drinking, according to drinks/day or gr of ethanol/day), as defined in the included studies. Alternatively, for the drinking levels will be defined: 1 unit as 8 g or 10ml of ethanol, and light as ‹ 2 units per day; moderate as 2-3 units per day; heavy as >= 4 units per day, in accordance with standard recommended alcohol allowance guidance (UK). If a study does not report the alcohol consumption levels, a dichotomy of any alcohol consumption versus non- alcohol consumption will be used. We will exclude studies on special populations (alcoholics, patients HBV/HCV infected) and studies referred only on specific types of alcoholic beverages.

21 Comparator(s)/control

Where relevant, give details of the alternatives against which the main subject/topic of the review will be compared (e.g. another intervention or a non-exposed control group).
The comparison group will be adults who are not exposed to alcohol, or where drinking levels are considered within the included studies. Also, the comparison groups will be adults who are exposed to lower levels of alcohol consumption.

22 Types of study to be included

Give details of the study designs to be included in the review. If there are no restrictions on the types of study design eligible for inclusion, this should be stated.
We will include longitudinal or cohort studies which have assessed the effect of alcohol on the outcomes of interest. Where there is limited longitudinal evidence for particular outcomes, we will also include case control studies.

23 Context

Give summary details of the setting and other relevant characteristics which help define the inclusion or exclusion criteria.

24 Primary outcome(s)

Give the most important outcomes.
We will include all studies which assess the effect of alcohol on the incidence of the disease. Diagnosis of incidence of disease from death certificates will also be eligible for inclusion. We will also assess the effect of alcohol on specific cancer: Upper aerodigestive tract cancers (oral cavity, larynx,pharynx, esophagus), colorectum, liver, female breast, prostate, lung, bladder, pancreatic, endometrial, ovarian, skin cancer, renal cell, small intestine and leukemia. We will examine the association between alcohol and severe lung diseases: pneumonia, tuberculosis, acute respiratory distress syndrome, chronic obstructive pulmonary disease, asthma and sleep apnoea.
Give information on timing and effect measures, as appropriate.
We will include all relative effect measures, for example Hazard Ratios, Odds Ratios, risk Ratios.

25 Secondary outcomes

List any additional outcomes that will be addressed. If there are no secondary outcomes enter None.
None

Give information on timing and effect measures, as appropriate.

26 **Data extraction (selection and coding)**

Give the procedure for selecting studies for the review and extracting data, including the number of researchers involved and how discrepancies will be resolved. List the data to be extracted.

Two reviewers will examine independently both the titles and the abstracts that have identified by electronic search in order to select the relevant included articles. Then the full text of potentially eligible articles will be searched and read by the reviewers, checking each paper against the inclusion criteria. Any disagreements will be resolved through discussion with a third reviewer. Two reviewers will independently screen all the studies and abstracted the following information in a piloted and standard format: study design, time period, participants, exposures, study setting and outcomes related to cancer and severe lung diseases. Disagreements regarding eligibility will be resolved through discussion or with a third reviewer.

27 **Risk of bias (quality) assessment**

State whether and how risk of bias will be assessed, how the quality of individual studies will be assessed, and whether and how this will influence the planned synthesis.

Two reviewers will independently conduct the quality assessment and the risk of bias of the included studies using the Newcastle-Ottawa Scale for longitudinal and cohort studies and the Assessment of Multiple systematic Reviews (AMSTAR) Scale for systematic reviews. Disagreements between the review authors over the risk of bias in particular studies will be resolved by discussion, with involvement of a third review author where necessary.

28 **Strategy for data synthesis**

Give the planned general approach to be used, for example whether the data to be used will be aggregate or at the level of individual participants, and whether a quantitative or narrative (descriptive) synthesis is planned. Where appropriate a brief outline of analytic approach should be given.

We will conduct a meta-analysis to synthesize the data. We will extract specific effect measures for the association between alcohol consumption and the risk of the disease (cancer or lung infection). Risk estimates will be reported as odds ratios (OR), risk ratios (RR), hazard ratios (HR) or incidence rate ratios (IRR) with 95% confidence intervals (CI). We will use a random effect meta-analytic model to calculate summary estimates of similar studies. The I2 statistics will be used to evaluate heterogeneity and forest plots used for the graphic investigation of the heterogeneity. Also, funnel plots will be used to visually assess evidence of publication bias. We will also conduct sensitivity analysis by excluding each study at a time from the meta-analysis to assess the influence of individual studies on the pooled effect measure. All the statistical analyses will be carried out using the STATA software and Review Manager 5.3 version software.

29 **Analysis of subgroups or subsets**

Give any planned exploration of subgroups or subsets within the review. 'None planned' is a valid response if no subgroup analyses are planned.

If the necessary data are available,we will perform subgroup and meta-regression analyses to assess reasons for heterogeneity between the studies, based on the geographical area (studies conducted in Europe compared to the rest of the world), sex, and whether the results were adjusted for confounding.

Review general information

30 **Type and method of review**

Select the type of review and the review method from the drop down list.

Epidemiologic, Systematic review

31 **Language**

Select the language(s) in which the review is being written and will be made available, from the drop down list. Use the control key to select more than one language.

English

Will a summary/abstract be made available in English?

Yes

32 **Country**

Select the country in which the review is being carried out from the drop down list. For multi-national collaborations select all the countries involved. Use the control key to select more than one country.

England

33 **Other registration details**

Give the name of any organisation where the systematic review title or protocol is registered together with any unique identification number assigned. If extracted data will be stored and made available through a repository such as the Systematic Review Data Repository (SRDR), details and a link should be included here.

34 **Reference and/or URL for published protocol**

Give the citation for the published protocol, if there is one.

Give the link to the published protocol, if there is one. This may be to an external site or to a protocol deposited with CRD in pdf format.

I give permission for this file to be made publicly available

Yes

35 Dissemination plans

Give brief details of plans for communicating essential messages from the review to the appropriate audiences.

A qualitative evaluation will be conducted to gain an understanding of the public's beliefs of the harms of alcohol on health. All the findings from these reviews will be used to develop a comprehensive website, where the target audiences are the academic community, professionals and general public. This website will also be evaluated by the users to ensure it is understandable and accessible to the aforementioned targeted groups

Do you intend to publish the review on completion?

Yes

36 Keywords

Give words or phrases that best describe the review. (One word per box, create a new box for each term)

systematic review
meta-analysis
alcohol
cancer
lung diseases

37 Details of any existing review of the same topic by the same authors

Give details of earlier versions of the systematic review if an update of an existing review is being registered, including full bibliographic reference if possible.

38 Current review status

Review status should be updated when the review is completed and when it is published.

Ongoing

39 Any additional information

Provide any further information the review team consider relevant to the registration of the review.

40 Details of final report/publication(s)

This field should be left empty until details of the completed review are available.
Give the full citation for the final report or publication of the systematic review.
Give the URL where available.

review only

PRISMA Checklist

Section/topic	#	Checklist item	Reported on page #
**TITLE**			
Title	1	Identify the report as a systematic review, meta-analysis, or both.	1
**ABSTRACT**			
Structured summary	2	Provide a structured summary including, as applicable: background; objectives; data sources; study eligibility criteria, participants, and interventions; study appraisal and synthesis methods; results; limitations; conclusions and implications of key findings; systematic review registration number.	1
**INTRODUCTION**			
Rationale	3	Describe the rationale for the review in the context of what is already known.	1
Objectives	4	Provide an explicit statement of questions being addressed with reference to participants, interventions, comparisons, outcomes, and study design (PICOS).	1,2
**METHODS**			
Protocol and registration	5	Indicate if a review protocol exists, if and where it can be accessed (e.g., Web address), and, if available, provide registration information including registration number.	2
Eligibility criteria	6	Specify study characteristics (e.g., PICOS, length of follow-up) and report characteristics (e.g., years considered, language, publication status) used as criteria for eligibility, giving rationale.	2
Information sources	7	Describe all information sources (e.g., databases with dates of coverage, contact with study authors to identify additional studies) in the search and date last searched.	2
Search	8	Present full electronic search strategy for at least one database, including any limits used, such that it could be repeated.	2
Study selection	9	State the process for selecting studies (i.e., screening, eligibility, included in systematic review, and, if applicable, included in the meta-analysis).	2
Data collection process	10	Describe method of data extraction from reports (e.g., piloted forms, independently, in duplicate) and any processes for obtaining and confirming data from investigators.	3
Data items	11	List and define all variables for which data were sought (e.g., PICOS, funding sources) and any assumptions and simplifications made.	3

For peer review only

Section/topic	#	Checklist item	Reported on page #
Risk of bias in individual studies	12	Describe methods used for assessing risk of bias of individual studies (including specification of whether this was done at the study or outcome level), and how this information is to be used in any data synthesis.	3
Summary measures	13	State the principal summary measures (e.g., risk ratio, difference in means).	3
Synthesis of results	14	Describe the methods of handling data and combining results of studies, if done, including measures of consistency (e.g., I^2) for each meta-analysis.	3,4
**Section/topic**	**#**	**Checklist item**	**Reported on page #**
Risk of bias across studies	15	Specify any assessment of risk of bias that may affect the cumulative evidence (e.g., publication bias, selective reporting within studies).	3
Additional analyses	16	Describe methods of additional analyses (e.g., sensitivity or subgroup analyses, meta-regression), if done, indicating which were pre-specified.	4
**RESULTS**			
Study selection	17	Give numbers of studies screened, assessed for eligibility, and included in the review, with reasons for exclusions at each stage, ideally with a flow diagram.	4
Study characteristics	18	For each study, present characteristics for which data were extracted (e.g., study size, PICOS, follow-up period) and provide the citations.	4,5,6
Risk of bias within studies	19	Present data on risk of bias of each study and, if available, any outcome level assessment (see item 12).	5
Results of individual studies	20	For all outcomes considered (benefits or harms), present, for each study: (a) simple summary data for each intervention group (b) effect estimates and confidence intervals, ideally with a forest plot.	7
Synthesis of results	21	Present results of each meta-analysis done, including confidence intervals and measures of consistency.	7
Risk of bias across studies	22	Present results of any assessment of risk of bias across studies (see Item 15).	7
Additional analysis	23	Give results of additional analyses, if done (e.g., sensitivity or subgroup analyses, meta-regression [see Item 16]).	7, 8
**DISCUSSION**			
Summary of evidence	24	Summarize the main findings including the strength of evidence for each main outcome; consider their relevance to key groups (e.g., healthcare providers, users, and policy makers).	8
Limitations	25	Discuss limitations at study and outcome level (e.g., risk of bias), and at review-level (e.g., incomplete retrieval of identified research, reporting bias).	8

For peer review only

Conclusions	26	Provide a general interpretation of the results in the context of other evidence, and implications for future research.	9
**FUNDING**			
Funding	27	Describe sources of funding for the systematic review and other support (e.g., supply of data); role of funders for the systematic review.	9

For peer review only
